# Drought modulates ozone stress through BVOCs, antioxidant defenses, and metabolic responses in a tropical tree

**DOI:** 10.1007/s00425-026-05033-8

**Published:** 2026-07-20

**Authors:** Fernanda Anselmo-Moreira, Alice Claude, Alex do Nascimento, Bruno Ruiz Brandão da Costa, Ivan Hurtado-Caceres, Manon Rocco, Michael Staudt, Adalgiza Fornaro, Agnès Borbon, Cláudia Maria Furlan, Silvia Ribeiro de Souza

**Affiliations:** 1 Departamento de Uso Sustentável de Recursos Naturais, Unidade Jardim Botânico, Instituto de Pesquisas Ambientais, São Paulo, SP 04301-002 Brazil; 2 Univ Paris Est Creteil, CNRS, Sorbonne Université, INRAE, IRD, IEES-Paris, Institut D’Ecologie et des Sciences de L’Environnement de Paris, F-94010, Créteil, France; 3 Centro de Ciências Naturais e Humana, Universidade Federal do ABC, Santo André, SP 09210-580 Brazil; 4 Departamento de Botânica, Instituto de Biociências, Universidade de São Paulo, Rua do Matão, 277, São Paulo, SP 05508-090 Brazil; 5 Departamento de Ciências Atmosféricas, Instituto de Astronomia, Geofísica e Ciências Atmosféricas, Universidade de São Paulo, São Paulo, SP 05508-090 Brazil; 6 Institut Méditerranéen d’Océanologie (MIO), Aix Marseille Univ, Université de Toulon, CNRS, IRD, MIO, Marseille, France; 7 CEFE, CNRS, EPHE, IRD, Univ Montpellier, Montpellier, France; 8 Laboratoire de Météorologie Physique, Université Clermont Auvergne, LaMP/CNRS, 6016, Clermont-Ferrand, France

**Keywords:** Abiotic stress, Ascorbate–glutathione cycle, Combined stress, Gas exchange, Metabolomics, Plant volatiles, Sesquiterpenes

## Abstract

**Main conclusion:**

Water limitation attenuated ozone-induced oxidative stress in Eugenia uniflora, maintaining physiological stability and promoting targeted biogenic volatile organic compounds (BVOCs) and metabolic adjustments rather than large-scale disruption.

**Abstract:**

Tropical forests are increasingly exposed to multiple environmental stressors, yet the combined effects of drought and ozone on native tropical tree species remain poorly understood. We investigated the individual and interactive effects of water limitation and ozone exposure on *Eugenia uniflora* saplings using an integrated physiological, biochemical, metabolomic, and biogenic volatile organic compound (BVOC) emission approach. Saplings were subjected to four treatments: control, drought, ozone fumigation, and combined drought-ozone. Drought was imposed by withholding irrigation for ten days, while ozone exposure (80–120 ppb) was applied for six days, reaching a cumulative AOT40 of 930.7 ppb h. Despite reductions in soil moisture, no significant changes were observed in leaf relative water content, gas exchange, or photosynthetic pigment, indicating high physiological resilience. Ozone exposure alone induced depletion of antioxidant pools, reducing ascorbate and glutathione concentrations, while redox ratios remained stable. BVOC emissions comprised 15 compounds across five chemical classes, dominated by oxygenated sesquiterpenes and sesquiterpenes. Although total emission rates did not differ among treatments, multivariate analyses revealed shifts in BVOC composition, including methyl salicylate and β-ocimene under water limitation and farnesane under combined stress. Metabolomic profiling revealed coordinated adjustments in sugars, amino acids, and oleanolic acid associated with ozone exposure. Notably, antioxidant depletion observed under ozone alone was not detected under combined stress, suggesting a non-additive interaction between water limitation and ozone. Overall, *E. uniflora* maintained physiological stability while adjusting volatile emissions and metabolic pathways under concurrent stress, providing new insights into the mechanisms governing drought–ozone interactions in tropical trees.

**Supplementary Information:**

The online version contains supplementary material available at 10.1007/s00425-026-05033-8.

## Introduction

Climate change is reshaping environmental conditions worldwide, leading to an unprecedented increase in the frequency, intensity, and co-occurrence of abiotic stresses such as drought, heatwaves, wildfires and floods (Marx et al. 2021). Because climate also affects air quality, it can alter the dynamics of atmospheric pollutants such as ozone (O₃), one of the most harmful pollutants to both human health and vegetation (Jacob and Winner 2009). Accordingly, increasing attention has been drawn to the interplay between drought events and O₃ pollution, as drought can exacerbate surface O₃ concentrations and prolong exposure periods (Demetillo et al. 2019; Lin et al. 2020). Together, these stressors can impair plant performance, ecosystem functioning, and biodiversity, particularly in tropical and subtropical regions (Zandalinas et al. 2021; Emmerichs et al. 2024). The risk may be especially high in urban and periurban environments, where native species persist in highly fragmented forest remnants under multiple anthropogenic pressures.

Drought impairs plant water status, photosynthesis, and metabolic homeostasis, often leading to oxidative stress (Gupta et al. 2020; Sharma et al. 2020). Likewise, tropospheric O₃ is a major phytotoxic pollutant that enters through the stomata, promotes reactive oxygen species (ROS) formation, and affects photosynthetic, biochemical, and volatile emission responses, with substantial variation among species (Ainsworth et al. 2012; Moura et al. 2022a).

One of the main biochemical mechanisms involved in the ROS detoxification in plants is the ascorbate–glutathione (AsA–GSH) cycle, a central component of the cellular antioxidant network. This pathway operates through the coordinated action of enzymes such as ascorbate peroxidase (APX), monodehydroascorbate reductase (MDHAR), dehydroascorbate reductase (DHAR), and glutathione reductase (GR), which together maintain the redox balance between reduced and oxidized forms of ascorbate and glutathione (Souza et al. 2013). Through this cycle, hydrogen peroxide (H₂O₂) generated under stress conditions is efficiently detoxified, helping to preserve cellular redox homeostasis. In addition to enzymatic antioxidant defenses, plants often respond to oxidative stress by adjusting their secondary metabolism and altering the emission of biogenic volatile organic compounds (BVOCs), including terpenoids and green leaf volatiles (GLVs). These compounds may contribute to oxidative stress mitigation, cellular signaling, and plant–environment interactions. Both drought and O_3_ exposure are known to influence the dynamics of the AsA–GSH system, BVOC emissions, and broader metabolomic profiles. Therefore, assessing changes in antioxidant metabolism together with BVOC emissions and metabolomic shifts provides important insights into the biochemical strategies used by plants to cope with multiple environmental stressors (Yu and Blande 2022).

Although the individual effects of both stressors are well documented, their combined impacts can be complex and difficult to predict (Dusart et al. 2019). This complexity has been recognized in both controlled and field studies, which show that water availability can modulate plant responses to O_3_, from growth effects under contrasting irrigation regimes (Bungener et al. 1999) to long-term biomonitoring evidence of lower visible O_3_ injury under drought conditions in forest plants (Smith 2012). Recent field experiments further show that drought does not necessarily alleviate O_3_ impacts under realistic exposure conditions (Martin et al. 2025). These interactions are particularly relevant under climate change scenarios (Otu-Larbi et al. 2020), yet their effects on tropical native species remain poorly understood. Integrated studies assessing both physiological and biochemical responses to combined O_3_ and drought stress also remain scarce, limiting our understanding of plant adjustment under increasingly common multiple-stress conditions.

A relevant tropical native species in this context is *Eugenia uniflora* L. (Myrtaceae), commonly known as pitangueira, a neotropical tree native to South America and widely distributed in southern and southeastern Brazil. This species has ecological and economic importance, being commonly used in urban landscaping, restoration of degraded areas, and domestic orchards. It also has recognized medicinal potential owing to bioactive compounds with antimicrobial, antioxidant, and anti-inflammatory properties (Moura et al. 2018; Fidelis et al. 2022). Despite its broad use in urban environments and the general assumption of stress tolerance, experimental evidence suggests that *E. uniflora* may be physiologically sensitive to chronic tropospheric O₃ exposure. In a recent study conducted under an O₃-FACE system, seedlings exposed for 75 days to elevated O₃ concentrations (approximately twice ambient levels) showed significant physiological and biochemical alterations, including reduced photosynthetic performance, changes in antioxidant defenses, and declines in soluble sugars, amino acids, and fatty acids, even in the absence of visible foliar injury (Engela et al. 2021). These findings indicate that O₃ impacts on *E. uniflora* may occur at sub-visible levels and depend on exposure regime, highlighting the need to assess the species under different O_3_ scenarios, including short-term exposure.

In this context, the present study investigated how drought and O₃ interact to influence the physiological performance, antioxidant metabolism, and biogenic volatile organic compound (BVOC) emissions of *Eugenia uniflora* saplings. Specifically, we addressed the following question: how does the simultaneous occurrence of water limitation and short-term O₃ exposure affect plant physiological stability, defined here as the maintenance of key physiological functions, including leaf water status and gas exchange, and shape metabolic responses in a tropical tree species? We hypothesized that even under a short exposure period, the combined occurrence of drought and O₃ could promote a synergistic amplification of stress responses, leading to more pronounced physiological and biochemical changes than those observed under each stressor applied individually. Also, we expected that water limitation-induced changes in plant water status and metabolic adjustment could modulate O₃ uptake, leading to distinct physiological and biochemical responses under combined stress compared with those observed under each stressor applied individually. Our findings contribute to a deeper understanding of how key native species respond to concurrent environmental pressures that are increasingly frequent under climate change. This knowledge is essential for improving conservation strategies, guiding urban forest management, and predicting the resilience of native flora in rapidly changing environments.

## Material and methods

### Plant material and experimental design

Twenty *E. uniflora* saplings were obtained from the Bioflora nursery (Piracicaba, São Paulo, Brazil) and transplanted into 3-L pots containing a commercial substrate (Carolina Soil®; Pardinho, São Paulo, Brazil), composed of peat, perlite, vermiculite, organic residues, and NPK fertilizer. The plants were grown in a greenhouse with filtered air (free of O₃ and particulate matter) under controlled irrigation at the Environmental Research Institute of São Paulo, Brazil (IPA-SP). Prior to the experiment, each plant received a single application of 50 mL of Hoagland solution.

The experiment was conducted in two closed fumigation chambers at the Atmosphere-Plant Interaction Laboratory (LABIAP), IPA-SP, Brazil (Fig. S1). Each chamber consisted of a Teflon-coated stainless steel structure with an internal volume of 1 m^3^ and a controlled air circulation system. Air was distributed through a square manifold constructed from stainless steel tubing (20 mm internal diameter), positioned midway between the base and the top of the chamber to ensure homogeneous air mixing. One chamber received charcoal-filtered air (control conditions), while the other was supplied with O_3_-enriched air generated from pure oxygen using an O_3_ generator. O_3_ concentration inside the chamber was continuously monitored and manually adjusted to maintain the target levels (Souza and Pagliuso 2009). Minor fluctuations in O₃ concentration, including occasional peaks, were associated with the dynamics of O₃ generation and air mixing within the chamber, which are typical of controlled fumigation systems. The two chambers were identical in construction and were operated within the same laboratory under the same general experimental conditions and schedule. Photosynthetically active radiation (PAR) was periodically checked and adjusted to remain at approximately 700 µmol m⁻^2^ s⁻^1^. However, chamber-specific continuous records of temperature and relative humidity were not obtained during this experiment.

Four-month-old *E. uniflora* saplings were randomly allocated to four experimental groups: control (C), drought (D), O₃ (O), and combined stress (DO), with five plants per group (*n* = 5). All saplings underwent a three-day acclimation period in growth chambers prior to the application of stress treatments (Day 0), followed by a 10-day experimental period (from Day 1 to Day 10). At the start of the experiment, saplings were approximately 60–70 cm in height, with an average of 10–12 nodes and 120–130 leaves (Fig. S2).

Saplings assigned to O_3_ treatments (O and DO) were placed in the O_3_ chamber during fumigation periods, while control treatments (C and D) remained in the filtered-air chamber. Outside fumigation periods, plants were maintained under identical environmental conditions. During fumigation periods, a total of 10 saplings were placed in each chamber, comprising five well-watered and five drought-treated plants per chamber. Thus, water treatments were applied within each chamber, while O_3_ exposure was defined at the chamber level.

Control plants were exposed to filtered ambient air and received 50 mL of water daily. Drought was imposed as a short-term water limitation treatment by withholding irrigation for 10 days, beginning three days prior to the onset of O₃ fumigation and maintained throughout the experimental period, representing a short-term water limitation treatment. Ozone stress was applied by exposing plants to O₃ concentrations of 80–120 ppb for five hours per day (10 a.m. to 3 p.m.) over six consecutive days, with occasional peaks exceeding 200 ppb, as described by Moura et al. (2022a). O₃ exposure was quantified as accumulated exposure above 40 ppb (AOT40).

Figure S1 summarizes the experimental design and indicates when each analysis was performed. Briefly, soil moisture, visual leaf injury, gas exchange measurements, and BVOC emissions were assessed at the end of the experimental period (Day 10), between 08:00 and 14:00 h, when plants were metabolically active. Due to logistical constraints and the need to perform all measurements under comparable environmental conditions, leaf material for biochemical analyses (AsA–GSH cycle components, metabolomic profiling, and photosynthetic pigments) was collected on the following day (Day 11) within the same time window. The collected material was immediately frozen at −80 °C. Relative water content (RWC) was determined on the same day (Day 11), immediately after leaf sampling for biochemical analyses, using leaf discs collected from different leaves. Plants subjected to water limitation treatments (D and DO) were not rehydrated prior to measurements, and soil moisture remained reduced at the time of evaluation.

The chambers were illuminated for nine hours daily (8 a.m. to 5 p.m.) using full-spectrum LEDs designed for indoor cultivation (Quantum board LM301H + Deep red 660 nm + UV + IR), providing a PAR of 700 µmol m^−2^ s^−1^ at canopy level. Air temperature and humidity in the laboratory environment outside the chambers were recorded three times per day (8 a.m., 12 p.m., and 3 p.m.) using a probe thermo-hygrometer (Simpla, TH01). Soil volumetric water content (% v/v) was monitored every two days using a ThetaProbe sensor (ML2x) connected to a moisture meter (HH2; Delta-T Devices, Burwell, UK), with measurements taken in each pot. Maximum soil moisture values (~ 40–50%) reflect the high porosity of the substrate used and are consistent with typical volumetric water content under field capacity conditions.

### Visible leaf injury

Visible leaf injury was assessed at the end of the stress treatment. For each saplings, the number of leaves exhibiting visible symptoms, such as dry tips and/or small spots with brown, red, or other pigmentation, was recorded as an absolute count.

### Photosynthetic pigments

To determine photosynthetic pigment content (chlorophyll *a* (Chl *a*), chlorophyll *b* (Chl *b*), total chlorophyll (TChl), Chl *a*/Chl *b*, and carotenoids), 50 mg of fresh frozen leaves were extracted with 1.5 mL of 96% ethanol in the dark for 2 h at 4 °C. After extraction, the samples were centrifuged at 13,700 g at 4 °C for 15 min. Then, 250 μL of the supernatant was transferred to the wells of a 96-well microplate. Absorbance readings were performed in triplicate using a Synergy H^1^ microplate reader (BioTek, Inc.), scanning from 200–750 nm.

The calculation of photosynthetic pigment concentrations, using 96% ethanol as the solvent, was based on the equations described by Lichtenthaler and Wellburn (1983), as follows:

1 $$Chla\, = \,13.95\, \times \,A_{665} {-}6.88\, \times \,A_{649}$$

2 $$Chlb\, = \,24.96\, \times \,A_{649} {-}7.32\, \times \,A_{665}$$

3 $$TChl\, = \,Chla\, + \,Chlb$$

4 $$Carotenoids\, = \,\left( {1000\, \times \,A_{470} {-\!\!-}2.05\, \times \,Chla{-}114.8\, \times \,Chlb} \right)/245$$

where 'A' represents the absorbance at the specified wavelength.

Absorbance readings were adjusted for the optical path length *h* (cm) using the following equation (Eq. 5):

5 $$h = \frac{3V}{{\pi \left( {R^{2} + Rr + r^{2} } \right)}}$$

where *V* corresponds to the volume added to the microplate well (250 µL), while *R* (3.45 mm) and *r* (3.20 mm) represent the radii of the larger and smaller bases of the well, respectively.

### Relative water content (RWC)

RWC was determined using the fresh mass (FM), turgid mass (TM), and dry mass (DM) of five 7 mm leaf discs collected from different leaves (González and González-Vilar 2001). To obtain TM, the leaf discs were placed in vials containing deionized water and kept in the dark for 24 h at 4 °C, then gently blotted dry before weighing. RWC was calculated using the following formula (Eq. 6):

6 $$RWC = \left( {\frac{FM - DM}{{TM - DM}}} \right) \times 100$$

### Gas exchange

Leaf gas exchange was measured on fully expanded mature leaves from all saplings. Measurements were taken one day before water withholding and at the end of the experimental period. Stomatal conductance to water vapor (*g*_sw_) and CO_2_ assimilation rate (*A*) were determined using a LI-6800 portable photosynthesis system (LI-COR Biosciences, Lincoln, NE, USA) equipped with a clear-top leaf chamber (6800-12A) with a 1 × 3 cm leaf area. During measurements, light was provided by an external full-spectrum LED system designed for indoor cultivation, supplying a PAR of 700 µmol m⁻^2^ s⁻^1^ at canopy level. Measurements were performed in the morning, between 08:30 and 10:30 h. Leaves were enclosed in the chamber and allowed to acclimate for 5 min under the following conditions: 400 ppm CO_2_, 25 °C leaf temperature, 60% relative humidity, and saturating light with a photosynthetic photon flux density (PPFD) of 700 µmol photons m^−2^ s^−1^. Five measurements were then taken from one leaf per sapling.

### Non-enzymatic antioxidant activity

Ascorbate and glutathione were analyzed using high-performance liquid chromatography coupled to a diode array detector (HPLC–DAD, LC1260; Agilent Technologies), equipped with an Agilent Eclipse Plus C-18 column (4.6 × 150 mm, 4.6 µm) at 25 °C, with a solvent flow rate of 1 mL min^−1^ and a 10 mm flow cell. The mobile phase consisted of water acidified with phosphoric acid (H_3_PO_4_, pH 2.3). An isocratic method was used, with four-minute runs for the analysis of the reduced forms of ascorbic acid and glutathione and five-minute runs for total ascorbic acid and total glutathione. Ascorbic acid and glutathione were monitored at 245 nm and 194 nm, respectively.

For sample preparation, 150 mg of frozen and ground leaves were homogenized in 2 mL of 6% metaphosphoric acid (HPO_3_) and 0.5 mM ethylenediaminetetraacetic acid disodium salt dihydrate (EDTA-Na_2_). The homogenate was centrifuged at 10,622 g for 15 min at 4 °C, and the supernatant was collected. To determine the reduced forms of ascorbic acid (AsA) and glutathione (GSH), 100 µL of the supernatant was diluted in 400 µL of the mobile phase, filtered (PTFE 0.45 µm), and 50 µL was injected into the HPLC. To determine total ascorbic acid (AsAt) and total glutathione (GSHt), 100 µL of the supernatant was mixed with 20 µL of 0.16% dithiothreitol (DTT), prepared in 2 M sodium phosphate buffer (pH 7.0), and 10 µL of 45% potassium phosphate (K_2_HPO_4_). The mixture was kept in an ice bath, in the dark, for 20 min, after which the reaction was stopped by adding 20 µL of 2 M phosphoric acid. The material was then diluted in 350 µL of water, filtered (PTFE 0.45 µm), and 50 µL was injected into the HPLC (Alves et al. 2020; Sala-Carvalho et al. 2022).

Ascorbic acid and glutathione contents were determined by comparison with standard curves of ascorbic acid (0.5–200 µM) and glutathione (0.2–10 µM), analyzed under identical conditions. Results were expressed as micrograms of ascorbic acid or glutathione per gram of fresh mass (µg g^−1^ FM). Both reduced and total pools were quantified, and redox status was expressed as the ratios AsA/AsAt and GSH/GSHt.

### Biogenic volatile organic compounds (BVOC) sampling and analysis

Before BVOC collection, the plants were transferred to the laboratory and acclimated for one hour under a PAR level of 700 µmol m^−2^ s^−1^. The apical branch of *E. uniflora* saplings was then enclosed in Teflon bags (25 × 55 cm), previously conditioned at 100 °C for 1 h, and gently sealed with a Teflon cable tie. Volatiles were collected using Tenax TA adsorbent cartridges (100 mg, 60/80 mesh; Supelco, Bellefonte, PA, USA). One end of each cartridge was fixed at the top corner of the Teflon bag, while the other end was connected to an individual suction pump (SKS, USA), operating at 225 mL min^−1^. The incoming air was pre-filtered using an active charcoal and silica system and pumped using an oil-free compressor (Schulz, Joinville, Santa Catarina, Brazil) at a flow rate of approximately 1.2 L min^−1^. BVOC sampling lasted for 60 min, and all collections were performed between 9 a.m. and 1 p.m. (Moura et al. 2022a).

BVOC trapped in the Tenax TA cartridges were thermally desorbed by an automatic thermal desorption system (ATD650, Perkin Elmer) and analyzed by gas chromatography coupled to a mass spectrometer (GC–MS, Agilent 7890B-Agilent 5977 A), equipped with an HP-5 capillary column (50 m × 0.2 mm i.d. × 0.5 µm of film thickness). Mass spectrometric detection was performed using electron ionization (EI) at 70 eV in a full-scan mode (range of 50–600 m/z). Thermal desorption was performed with nitrogen gas assistance at 250 °C for 5 min, with a transfer temperature of 200 °C, a heating rate of 40 °C s⁻^1^, and a cryofocusing injection at − 30 °C. After desorption, BVOCs were injected into the column with helium as a carrier gas. The oven temperature program was set as follows: the initial temperature was 46 °C, held for 5 min, then increased at 5 °C min^−1^ to 210 °C, held for 20 min, and finally ramped at 10 °C min^−1^ to 250 °C, where it was maintained for 5 min (Araújo et al. 2025). Compound annotation was performed by comparing the sample mass spectra with those in the Wiley/NIST chemical library and with commercially available pure standards analyzed under the same conditions. Additionally, a blank (cartridge exposed to the same sampling and analysis conditions but without saplings) was included to detect and exclude any background contamination. After BVOC collection, the leaves enclosed in Teflon bags were oven-dried at 60 °C for 72 h with forced ventilation.

BVOC quantification was carried out using external calibration curves (R^2^ > 0.94 in all cases; Sigma-Aldrich, St. Louis, MO, USA), with concentrations ranging from 10 to 50 ng per compound. When authentic standards were not available, emission rates were estimated based on calibration curves of structurally related compounds. Accordingly, 3-carene was used to estimate isoprene and non-oxygenated monoterpenes (MTs), while methyl salicylate, humulene, and farnesol were used as reference compounds for salicylates, non-oxygenated sesquiterpenes (SQTs), and oxygenated sesquiterpenes (OSQTs), respectively.

The emission rate (*ER*) of each BVOC was calculated using the following equation:

$$ERi=\frac{F x Ci}{M}$$

where *F* is the flow rate at the inlet of the Teflon bag (L h⁻^1^), *Ci* is the mass concentration of the BVOC in the sample (μg L^−1^), and *M* is the dry mass (g) of the enclosed leaves. ERs were expressed as µg gDM^−1^ h⁻^1^.

The BVOC dataset was preprocessed to ensure data quality and analytical consistency: compounds detected in fewer than three biological replicates within a given treatment group were excluded from that specific group but retained in groups where they were consistently detected (i.e., present in at least three biological replicates). Missing values were imputed using the MissForest algorithm, implemented in the R package missForest (Stekhoven and Bühlmann 2012). For multivariate analyses, compounds that were absent across all replicates of a given treatment group were assigned a near-zero value (0.0001) to prevent matrix sparsity, allow log transformation, and enable appropriate clustering procedures.

## Metabolomics by GC–MS

### GC–MS analysis

To conduct metabolomic profiling, 20 mg of freeze-dried and ground leaves were extracted using 500 μL of a chloroform/methanol/water mixture (12:5:1, by vol.), along with 60 μL of adonitol (0.2 mg mL^−1^, as an internal standard for the polar phase). The mixture was incubated in a dry bath at 70 °C for 10 min, followed by centrifugation at 11,000 g. The supernatant was then collected, and 350 µL of ultrapure water was added, followed by centrifugation at 2200 g for 5 min. Then, 100 µL of the polar phase and 100 µL of the nonpolar phase were separately transferred to inserts and dried under vacuum (Engela et al. 2021, with modifications).

The polar phase underwent a two-step derivatization process: first, methoxyamination, in which the samples were reconstituted in 28 µL of methoxyamine hydrochloride in pyridine (20 mg mL^−1^) and heated at 37 °C for 1 h; second, trimethylsilylation, achieved by adding 48 μL of *N*-methyl-*N*-(trimethylsilyl)trifluoroacetamide (MSTFA, Sigma-Aldrich) and heating at 37 °C for 30 min. For the nonpolar phase, derivatization was performed by adding 25 µL of pyridine and 25 µL of *N*,*O*-bis(trimethylsilyl)trifluoroacetamide (BSTFA, Sigma-Aldrich), followed by heating at 70 °C for 1 h. Derivatization blanks were prepared for both polar and nonpolar phases to control for background signals.

After derivatization, the samples were analyzed using a GC–MS (Agilent 6580-Agilent 5975 C VL MSD), equipped with a HP-5 ms column (30 m × 0.25 mm × 0.25 μm film thickness). Helium was used as the carrier gas at a constant flow rate of 1 mL min^−1^. The injection volume was 1 µL, with a split ratio of 1:100. Mass spectrometric detection was performed by EI at 70 eV, operating in full-scan mode (50–600 m*/z* for polar phase and 50–700 m*/z* for nonpolar phase). For the polar phase analysis, the oven temperature program started with an initial hold at 70 °C for 5 min, followed by a ramp of 5 °C min^−1^ to 295 °C, then at 10 °C min^−1^ to 320 °C, with a final hold time of 2.5 min, totaling 55 min of runtime. For the nonpolar phase, the oven program began with a hold at 100 °C for 5 min, followed by a temperature increase of 5 °C min^−1^ to 320 °C, with a final hold of 8 min, totaling 57 min (Engela et al. 2021, with modifications).

### GC–MS data treatment

For data processing, both polar and nonpolar phases data were analyzed separately using the Global Natural Product Social Molecular Networking (GNPS) platform (Wang et al. 2016). This analysis included deconvolution, peak alignment, linear retention index (LRI) calculation, compound annotation, and molecular networking. The GNPS output included a feature table containing the peak area, retention time (RT), and balance score of each detected feature, and a separate annotation table containing the putative compound annotations and their corresponding retention indices. A minimum cosine similarity index of 0.70 was used for annotation. Compound identities were further validated using the mass spectral library NIST (National Institute of Standards and Technology) 2.0 and LRI comparisons. LRI values were calculated using C_8_ to C_40_ alkane standards analyzed under the same GC–MS conditions. To improve annotation confidence, LRI values were compared using three reference databases: the NIST digital library spectra (v2.0, 2008), the Golm Metabolome Database (GMD, http://gmd.mpimp-golm.mpg.de), and MassBank Europe (MB) (https://massbank.eu/MassBank/), using an acceptance window of 30 units.

To minimize non-biological variability and enhance feature reliability, data processing was performed prior to compound annotation. Features with a blank-to-sample abundance ratio exceeding 1:3 were excluded as potential contaminants or background noise (Pakkir Shah et al. 2025). Additionally, to ensure the inclusion of only high-quality deconvoluted spectra, features with a balance score equal to or greater than 65% were retained in the final sample feature list (Aksenov et al. 2021). Missing values were imputed using a below-LOD (limit of detection) strategy, replacing missing values with one-fifth of the lowest non-zero peak area intensity observed across the dataset. Missing values were assumed to represent compounds present below the detection limit (left-censored data), consistent with single-value imputation approaches commonly applied in metabolomics (Jin et al. 2018).

Annotation confidence levels were assigned according to the framework proposed by Valli et al. (2019). Level 1 annotations were confirmed using in-house reference standards, including both RT and fragmentation pattern. Level 2 annotations were based on matches with GNPS or spectral libraries, combined with LRI comparisons reported in the literature. Level 3 annotations corresponded to class-level identifications, supported by spectral similarity and clustering patterns observed in the molecular networking analysis. Level 4 annotations corresponded to unidentified features with no reliable structural information, which were retained as unknown. Results were expressed as the peak area of each annotated compound, and values were summed to obtain the relative abundance of each chemical class.

### Data analysis

Because O₃ exposure was applied at the chamber level, chamber and O₃ effects could not be statistically separated in the present experimental design. Therefore, statistical analyses were used to evaluate treatment-associated responses rather than to fully disentangle chamber-specific effects, and O₃-related responses were interpreted cautiously in light of this limitation. Significant differences among treatments for visible leaf symptoms, RWC, photosynthetic pigments, non-enzymatic antioxidants, and BVOCs were assessed using the Kruskal–Wallis test, followed by Dunn’s post hoc test with Benjamini–Hochberg (BH) correction for multiple comparisons. Metabolomic data were also analyzed using the Kruskal–Wallis test, and the resulting *P*-values were adjusted using the BH false discovery rate procedure due to the large number of detected metabolites.

In addition to significance testing, effect sizes were calculated for metabolomic data using the eta-squared statistic for the Kruskal–Wallis test (η^2^[H]), providing a measure of the global magnitude of treatment effects (Tomczak and Tomczak 2014). Effect sizes were interpreted according to conventional benchmarks (Cohen 1988; Lakens 2013), where values of 0.01, 0.06, and 0.14 indicate small, medium, and large effects, respectively. Pairwise effect sizes were further assessed using the Vargha and Delaney A statistic (VDA), which quantifies the probability that values from one group are larger than those from another. Pairwise effect sizes were interpreted using thresholds of 0.56, 0.64, and 0.71 for small, medium, and large effects, respectively (Vargha and Delaney 2000).

When comparisons between the beginning and end of the experiment were required (soil moisture and gas exchange), paired t-tests were applied. When necessary, data were log₁₀-transformed to meet assumptions of normality. For multivariate analyses, raw peak areas were treated as compositional data and transformed using a centered log-ratio (CLR) transformation implemented in the compositions package in R. The transformed data were subsequently autoscaled (mean-centering and division by the standard deviation) prior to multivariate analyses. Principal component analysis (PCA) was performed on the pre-processed data matrix to explore multivariate patterns among treatments using the FactoMineR package, with visualizations generated using factoextra and ggplot2. Global treatment effects were assessed by PERMANOVA based on Euclidean distances of the autoscaled data with 999 permutations, implemented using the vegan::adonis2 function.

To identify compounds contributing to treatment discrimination, Random Forest (RF) classification models were applied to both BVOC and metabolomic datasets using the randomForestExplainer package in R. Variable importance was evaluated based on the mean decrease in accuracy and mean decrease in Gini index, which quantify the contribution of each variable to model performance and group separation. Statistical significance of variable importance was assessed using permutation-based p-values, and compounds were considered significant according to the corresponding thresholds.

Hierarchical clustering analysis (HCA) was visualized using heatmaps to explore variation in BVOC profiles among treatments. Prior to clustering, peak areas were subjected to the same preprocessing steps applied in the other multivariate analyses. Heatmaps were generated using the ComplexHeatmap package in R. Hierarchical clustering was performed on the rows using Euclidean distance and the average linkage method. All analyses were performed in R version 4.5.1 within RStudio.

## Results

### Environmental conditions

During the experimental period, laboratory air temperature ranged from 21.6 to 24.2 °C and relative humidity from 67 to 72%. During O₃ fumigation, concentrations varied among exposure days, with daily mean values ranging from 29.0 to 88.7 ppb based on the available 5-min records. Occasional short-term peaks were observed, with a maximum recorded value of 221 ppb. After six days of fumigation, the cumulative AOT40 reached 930.7 ppb·h. Daily O₃ exposure profiles and summary values are provided in Suppl- Fig. S3 and Table S1, respectively.

### Visible leaf injury

After the 10-day treatment period, the occurrence of dry leaf tips (Fig. S4A) and small brown spots (Fig. S4B) on *E. uniflora* leaves was assessed. For dry tips, no statistically significant differences were detected among treatments (χ^2^(_3_) = 4.64, *P* = 0.200), although saplings from the water-deprived groups (D and DO) tended to show a higher number of affected leaves compared with the control (Table 1). Brown spot incidence differed significantly among treatments (χ^2^(3) = 9.73, *P* = 0.021), although Dunn's post hoc test did not detect significant pairwise differences (all *P* > 0.05). Interestingly, small brown spots were observed exclusively in saplings exposed to ozone fumigation (Table 1).

**Table 1:** Visible foliar symptoms (number of leaves with injuries), photosynthetic pigment contents (μg gFM^−1^; μg of photosynthetic pigments per g of fresh mass), and relative water content (%) in leaves of *Eugenia uniflora* L. seedlings exposed to drought and ozone stresses, alone or in combination

Parameters	Treatments				*P* value
	C	D	O	DO	
Symptoms					
Dry tips	14 ± 10	35 ± 31	7 ± 10	34 ± 24	0.2
Brown spots*	−	−	2 ± 2	2 ± 2	0.021
RWC	89.31 ± 3.53	90.13 ± 2.28	88.84 ± 4.38	87.85 ± 2.74	0.684
Photosynthetic pigments					
Chl a	394.58 ± 75.66	429.01 ± 123.59	436.97 ± 119.75	391.13 ± 53.96	0.925
Chl b	182.87 ± 36.20	201.68 ± 54.54	203.80 ± 52.60	186.76 ± 29.25	0.937
Chl a/Chl b	2.16 ± 0.11	2.12 ± 0.05	2.14 ± 0.06	2.10 ± 0.06	0.595
Total Chl	577.45 ± 111.13	630.68 ± 178.10	640.77 ± 172.18	577.89 ± 82.89	0.946
Carotenoids	78.84 ± 13.57	81.38 ± 17.11	85.22 ± 21.83	76.87 ± 9.85	0.972

Data are presented as the mean ± standard deviation

*C* control (*n*=5), *D* drought (*n*=5), *O* ozone (*n*=5), *DO* combined drought and ozone (*n*=5). *Chl* chlorophyll, *RWC* relative water content

Different letters indicate significant differences among treatments. Absence of letters denotes non-significant differences. Bold values indicate statistical significance at *P* ≤ 0.05.

Kruskal-Wallis test followed by Dunn’s post hoc test with Benjamini-Hochberg correction.

*The Kruskal-Wallis test showed a significant difference among groups (*P* < 0.05); however, post hoc multiple comparisons using Dunn's test did not identify any significant pairwise differences.

### Physiological responses

**Fig. 1:**
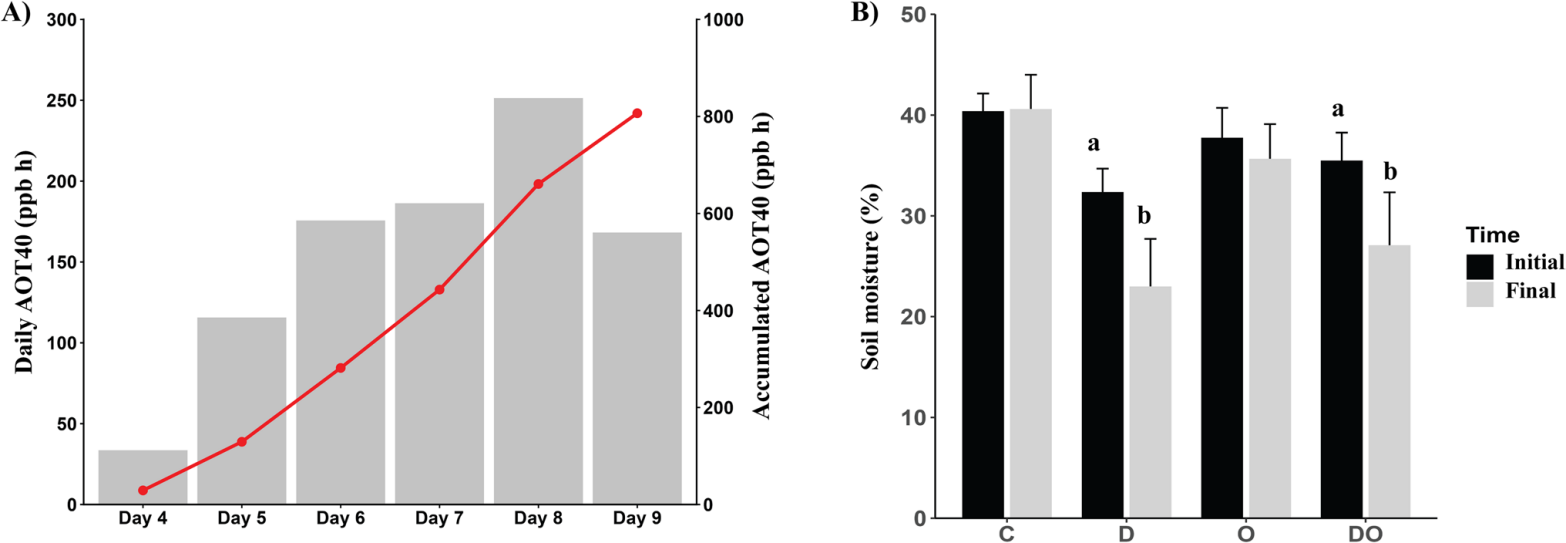
Ozone exposure and soil moisture dynamics in *Eugenia uniflora* saplings during the experiment. **A** Ozone conditions during the fumigation period (Days 4–9). Bars represent the daily AOT40 values for saplings exposed to ozone, and the red line represents the cumulative AOT40 across the fumigation period. AOT40: accumulated ozone exposure over the threshold of 40 ppb. **B** Soil volumetric water content (% v/v) in *Eugenia uniflora* saplings subjected to drought and ozone stresses, alone or in combination, comparing the initial and final measurements within each treatment. Data are presented as mean ± standard deviation (*n* = 5). *C* control, *D* drought, *O* ozone, *DO* combined drought and ozone. Different letters indicate significant differences between initial and final (paired t-test, *P* < 0.05). Absence of letters denotes non-significant differences (*P* > 0.05)

Comparing the beginning of the experiment (before water withdrawal) with the end, soil moisture did not decrease significantly in the C (*P* = 0.897) or O (*P* = 0.363) groups. In contrast, a significant reduction was observed in the water-deprived groups D (*P* = 0.006) and DO (*P* = 0.003), corresponding to decreases of 29% and 24% in the D and DO groups, respectively (Fig. 1B). Despite the reduction in soil moisture, RWC was not significantly affected in the D, O or DO treatments (χ^2^(_3_) = 1.49; *P* = 0.684) (Table 1), indicating that leaf water status was maintained in *E. uniflora* saplings.

**Fig. 2:**
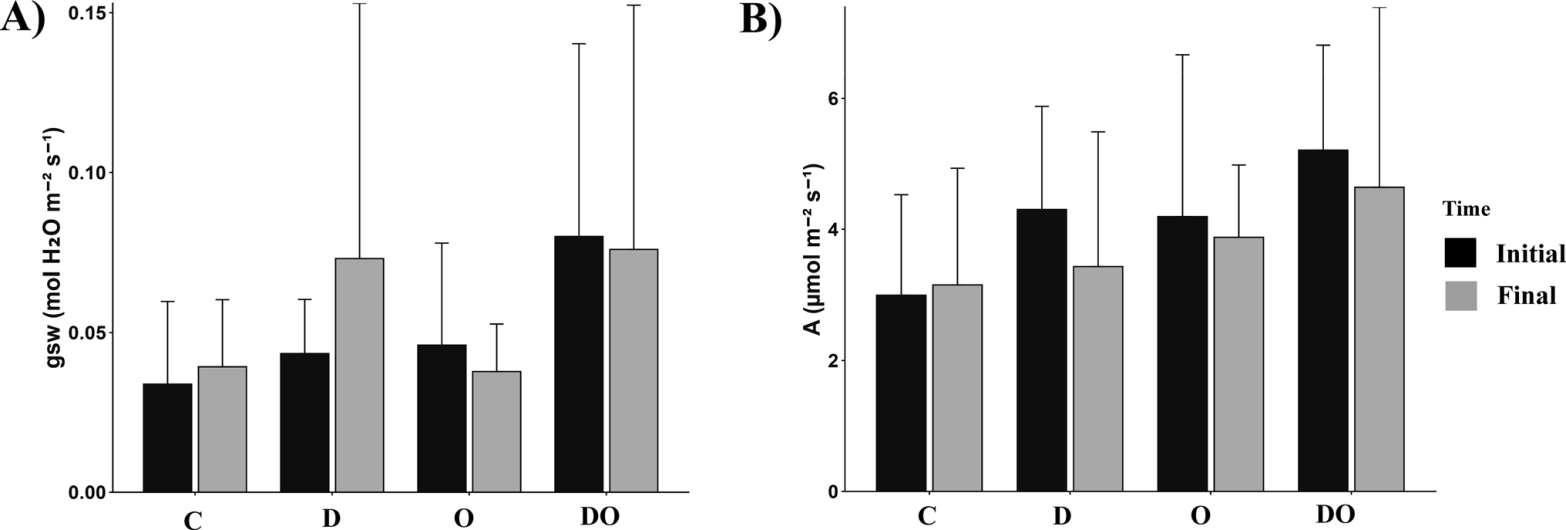
Gas exchange parameters in leaves of *Eugenia uniflora* saplings subjected to different treatments. **A** Stomatal conductance (*g*_sw_). **B** CO₂ assimilation rate (*A*). Data are presented as mean ± standard deviation (*n* = 5). *C* control, *D* drought, *O* ozone, *DO* combined drought and ozone. Statistical differences were assessed using paired t-tests comparing the initial and final measurements within each treatment. Different letters indicate significant differences between initial and final (*P* < 0.05). Absence of letters denotes non-significant differences (*P* > 0.05)

Similarly, gas exchange parameters remained stable throughout the experiment. When comparing values at the beginning and end of the exposure period, no significant differences were detected in *g*_sw_ (Fig. 2A) and *A* (Fig. 2B) within each treatment (all *P* > 0.05; Table S2). In addition, photosynthetic pigment contents did not differ among treatments. No significant differences were observed in Chl a (χ^2^(3) = 0.47, *P* = 0.925), Chl b (χ^2^(3) = 0.42, *P* = 0.937), TChl (χ^2^(3) = 0.37, *P* = 0.946), Chl *a*/Chl *b* (χ^2^(3) = 1.89, *P* = 0.595), or carotenoids (χ^2^(3) = 0.23, *P* = 0.972) (Table 1).

### Non-enzymatic antioxidant defenses

**Fig. 3:**
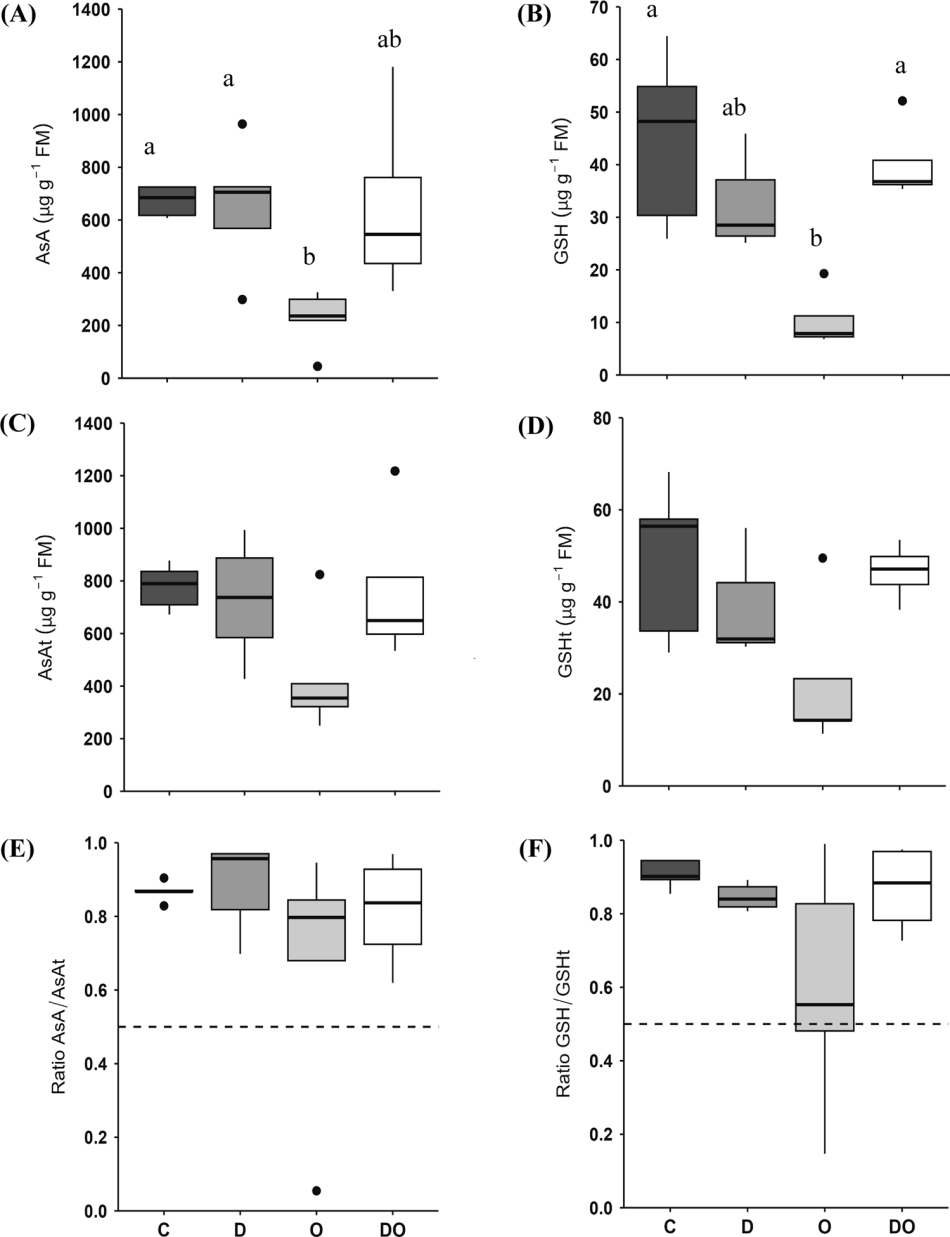
Non-enzymatic antioxidant content and redox status in leaves of *Eugenia uniflora* saplings subjected to different treatments. **A** Reduced ascorbic acid (AsA). **B** Reduced glutathione (GSH). **C** Total ascorbic acid (AsAt). **D** Total glutathione (GSHt). **E** AsA/AsAt ratio. **F** GSH/GSHt ratio. Antioxidant contents are expressed as µg g⁻^1^ fresh mass (FM). Boxplots represent the median (horizontal line), interquartile range (box), and minimum–maximum values (whiskers); dots indicate outliers. *C* control (*n* = 5), *D* drought (*n* = 5); *O* ozone (*n* = 5); DO combined drought and ozone (*n* = 4). Different letters indicate significant differences among treatments (*P* < 0.05; Kruskal–Wallis test followed by Dunn’s post hoc test with Benjamini–Hochberg correction). Absence of letters indicates no significant differences (*P* > 0.05)

The AsA leaf content differed significantly among treatments (χ^2^(3) = 9.54, *P* = 0.023), with O saplings showing significantly lower AsA concentrations than C (*P* = 0.035) and D (*P* = 0.040), corresponding to reductions of 66.5% and 65.5%, respectively (Fig. 3A). AsAt showed a similar decreasing trend under O₃ exposure, although this difference was not statistically significant (χ^2^(3) = 6.09, *P* = 0.107; Fig. 3C). Despite these changes, the AsA/AsAt ratio remained high (> 0.5) and did not differ among treatments (χ^2^(3) = 2.99, *P* = 0.393; Fig. 3E), indicating a preserved redox balance.

Similarly, GSH content was significantly affected by O₃ exposure (χ^2^(3) = 11.62, *P* = 0.009), with the O group showing lower GSH concentrations than both C (*P* = 0.012) and DO (*P* = 0.020), corresponding to reductions of 75.5% and 72.5%, respectively (Fig. 3B). GSHt followed the same trend, although no significant differences were detected among treatments (χ^2^(3) = 7.13, *P* = 0.068; Fig. 3D). The GSH/GSHt ratio also remained high (> 0.5) across all treatments, with no significant differences observed (χ^2^(3) = 4.16, *P* = 0.245; Fig. 3F). Full statistical results, including pairwise comparisons and adjusted *P*-values, are provided in Table S3.

### BVOCs emission profiles and treatment-driven changes

Fifteen BVOCs belonging to five distinct chemical classes were annotated, including isoprene, salicylates, non-oxygenated monoterpenes (MTs), SQTs, and OSQTs (Table 2). Overall, BVOC emission profiles were largely conserved across treatments, with a high degree of similarity in dominant compounds and relative class contributions among treatments (Table 2; Fig. S5A-B).

**Table 2:** Biogenic volatile organic compound (BVOC) emission rates (µg g⁻¹ DM h⁻¹) in leaves of *Eugenia uniflora* seedlings exposed to drought and ozone, alone or in combination

Chemical family	BVOC	Treatments			
		C	D	O	DO
BVOC total		**60.25 ± 56.36**	**230.81 ± 175.81**	**93.73 ± 105.88**	**72.28 ± 72.83**
*Isoprene*					
	Isoprene	0.14 ± 0.10	–	0.09 ± 0.04	−
*Salicylates*					
	Methyl salicylate	−	0.32 ± 0.08	−	0.27 ± 0.16
*MTs*					
	3-Carene	0.70 ± 0.27	–	−	−
	β-Ocimene	−	1.53 ± 0.28	−	1.96 ± 2.12
	**Total**	**0.70 ± 0.27**	**1.53 ± 0.28**	**−**	**1.96 ± 2.12**
*SQTs*					
	Alloaromadendrene	0.17 ± 0.14	−	0.07 ± 0.03	−
	Farnesane	0.22 ± 0.19	0.11 ± 0.02	0.09 ± 0.03	0.25 ± 0.17
	α-Copaene	0.26 ± 0.06	−	−	−
	β-Cadinene	0.30 ± 0.10	−	−	−
	β-Elemene	0.68 ± 0.62	1.43 ± 1.21	0.44 ± 0.39	0.62 ± 0.59
	γ-Elemene	1.58 ± 1.40	1.65 ± 1.97	−	−
	γ-Muurolene	−	0.20 ± 0.08	0.13 ± 0.09	−
	δ-Cadinene	−	0.16 ± 0.04	−	−
	δ-Elemene	0.74 ± 0.54	0.73 ± 0.71	0.35 ± 0.17	0.46 ± 0.40
	**Total**	**3.95 ± 1.84**	**4.28 ± 3.81**	**1.08 ± 0.48**	**1.33 ± 1.11**
OSQTs					
	Curzerene	50.38 ± 56.89	196.83 ± 150.67	63.75 ± 67.26	68.72 ± 70.14
	Germacrone	5.07 ± 3.37	27.84 ± 23.87	28.81 ± 41.17	−
	**Total**	**55.45 ± 56.07**	**224.68 ± 172.31**	**92.55 ± 105.49**	**68.72 ± 70.14**

Data are presented as the mean ± standard deviation

*C* control (*n* = 5), *D* drought (*n* = 5), *O* ozone (*n* = 5), *DO* combined drought and ozone (*n* = 4), *MTs*, non-oxygenated monoterpenes, *SQTs* non-oxygenated sesquiterpenes, *OSQTs* oxygenated sesquiterpenes—indicates below the detection limit or absent in the treatment

Bold values indicate total values

Despite the overall similarity in BVOC profiles, minor qualitative differences were detected among treatments. Of the 11 compounds detected in C saplings, four (curzerene, δ-elemene, β-elemene, and farnesane) were detected in all treatments. In contrast, methyl salicylate, β-ocimene, γ-muurolene, and δ-cadinene were absent from C plants; methyl salicylate and β-ocimene were associated with D and DO saplings, whereas δ-cadinene was detected only in D. Three compounds (3-carene, α-copaene, and β-cadinene) were exclusive to C saplings. Neither O nor DO treatments induced any unique BVOCs (Table 2).

Total BVOC emission rates did not differ significantly among treatments (Kruskal–Wallis, χ^2^(3) = 2.82, *P* = 0.420; Table 2). However, D saplings exhibited greater inter-individual variability in total emissions (Fig. S5C). The overall effect size was negligible (η^2^[H] ≈ 0.00), indicating that treatment explained virtually none of the observed variance. Among chemical classes, OSQTs represented the dominant fraction of BVOC emissions across all treatments, followed by SQTs and non-oxygenated monoterpenes (MTs) (Table 2; Fig. S5B).

**Fig. 4:**
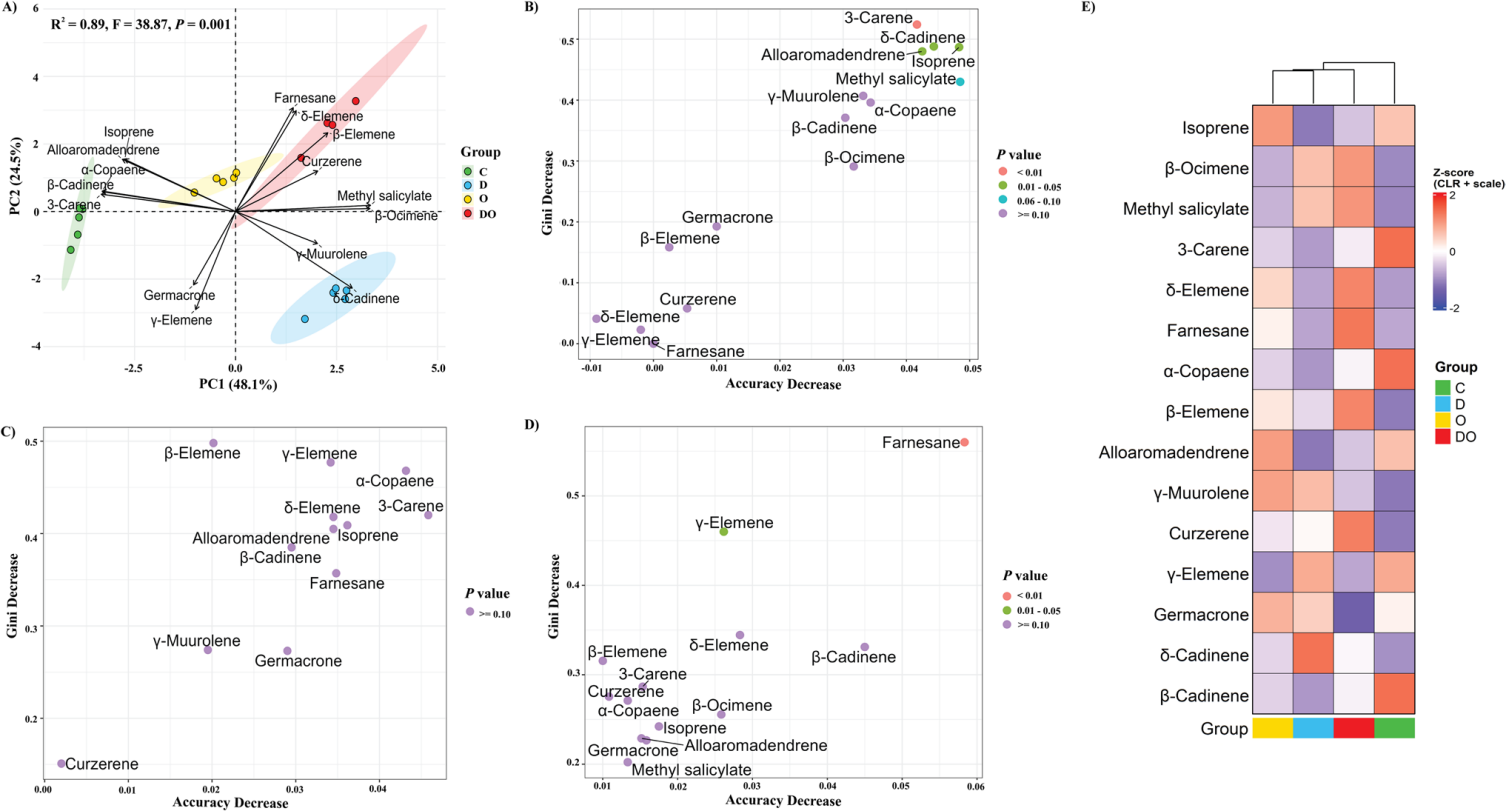
Multivariate analyses of biogenic volatile organic compound (BVOC) emission profiles in *Eugenia uniflora* saplings exposed to drought and ozone stress. **A** Principal component analysis (PCA) score plot showing the separation of treatments based on BVOC emission profiles, with vectors indicating the contribution of individual compounds. The PERMANOVA result assessing treatment effects is shown in the panel. **B** Random Forest variable importance plot for pairwise comparisons between C vs D. **C** Random Forest variable importance plot for pairwise comparisons between C vs O. **D** Random Forest variable importance plot for pairwise comparisons between C vs DO. In the Random Forest, colors indicate permutation-derived p-value thresholds indicating the statistical relevance of each variable. **E** Hierarchical clustering analysis (HCA) heatmap of BVOC emission profiles across treatments. Values represent Z-score standardized emission rates (LR + scale), and dendrograms indicate similarity among treatments. *C* control (*n* = 5), *D* drought (*n* = 5), *O* ozone (*n* = 5); DO, combined drought + ozone (*n* = 4)

Although BVOC emission rates and the dominant class contributions were similar among treatments, multivariate analyses revealed treatment-related differences in composition. PCA explained 72.6% of the total variance in BVOC emission profiles, with 48.1% and 24.5% captured by PC1 and PC2, respectively (Fig. 4A). The score plot indicated treatment-related structuring among C, D, O, and DO, and these patterns were supported by PERMANOVA (R^2^ = 0.89, F = 38.87, *P* = 0.001). Along PC1, C was clearly separated from the water-deprivation treatments (D and DO). This separation was driven by higher contributions of 3-carene, α-copaene, and β-cadinene, which were detected exclusively in C, whereas methyl salicylate and β-ocimene were detected only in D and DO (Fig. 4A, Fig. S6A, Table 2). PC2 further separated DO from D, mainly due to farnesane and δ-elemene (Fig. 4A; Fig. S6B).

To further identify the compounds contributing to group separation, RF analyses were performed by comparing each treatment with C. In the C vs. D comparison, 3-carene, δ-cadinene, isoprene, and alloaromadendrene emerged as the most important BVOCs, supporting the PCA results (Fig. 4B). In the C vs. O comparison, no BVOC significantly contributed to group separation (all *P* > 0.05; Fig. 4C). In contrast, in the C vs. DO comparison, farnesane and γ-elemene showed high importance values, consistent with their association with DO in the PCA (Fig. 4D).

Hierarchical clustering analysis (HCA) revealed two major groups, consistent with the PCA results: C formed a distinct cluster, whereas all stress treatments grouped together. Within the stress-treatment cluster, D and O clustered more closely with each other, while DO formed a more distinct subgroup (Fig. 4E).

### GC–MS characterization and treatment effects on the leaf metabolome of *E. uniflora*

In total, 349 features were detected across both phases, including 185 in the polar and 164 in the nonpolar phase. After manual curation and annotation, the final dataset comprised 54 metabolites in the polar phase and 61 in the nonpolar phase, accounting for 93% and 79% of total ion coverage, respectively. Among these, 18, 69, and 30 metabolites were assigned to confidence levels 1, 2, and 3, respectively (Tables S4 and S5). The polar phase was dominated by carbohydrates, polyols, amino acids, and organic acids, whereas the nonpolar phase comprised hydrocarbons, fatty acids, glycerolipids, sesquiterpenes, triterpenes, and phytosterols (Fig. S7A–B; Tables S6–S7).

**Fig. 5:**
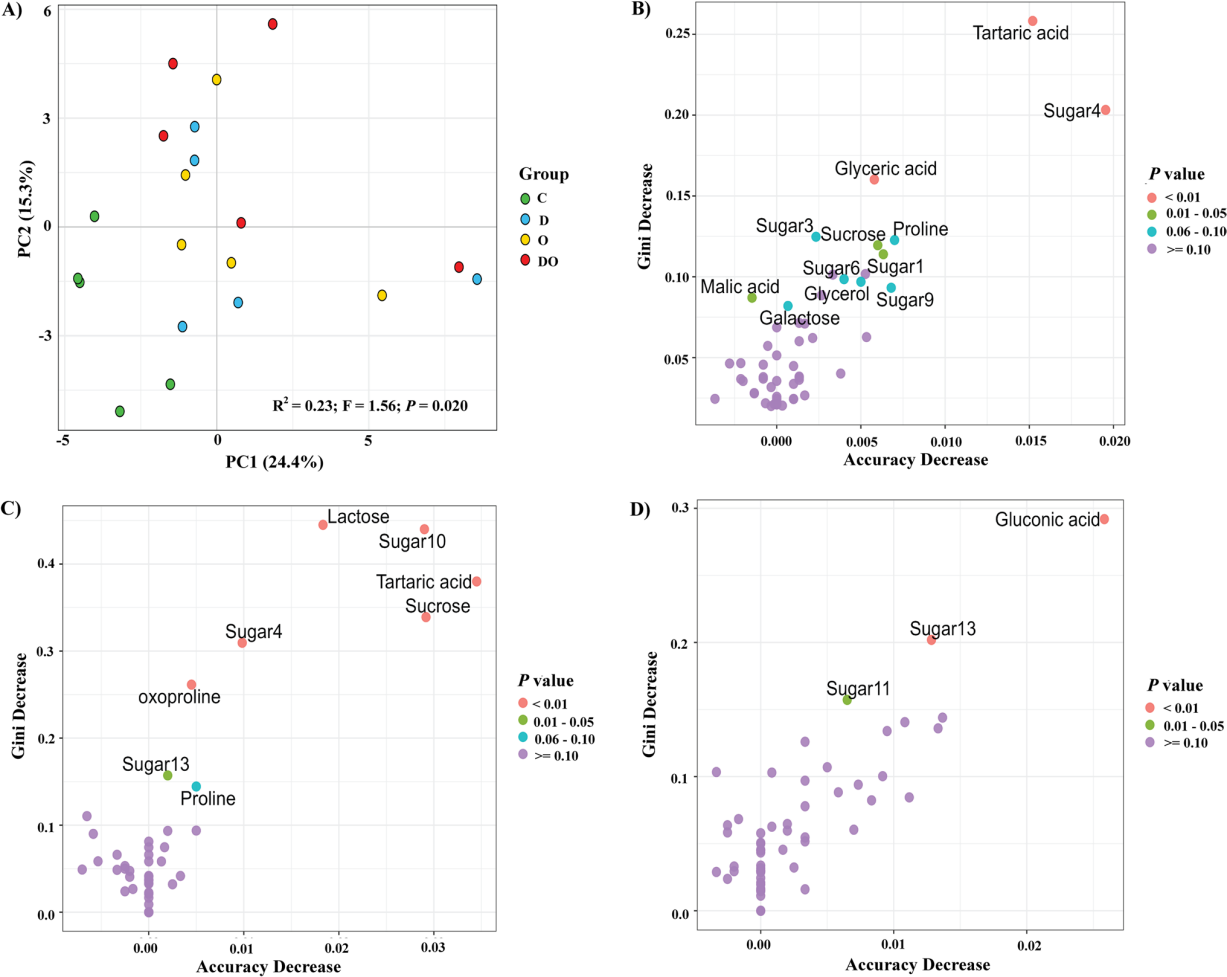
Multivariate analyses of the polar leaf metabolome of *Eugenia uniflora* saplings under drought and ozone stress. **A** PCA score plot of the based on annotated metabolites. **B–D** Random Forest variable importance plots for pairwise comparisons in the polar phase: C vs D (**B**), C vs O (**C**), and C vs DO (**D**). In the Random Forest plots, colors represent permutation-derived p-value thresholds indicating the statistical relevance of each variable. *C* control (*n* = 5), *D* drought (*n* = 5), *O* ozone (*n* = 5), *DO* combined drought + ozone (*n* = 5)

For the polar phase, PCA based on annotated compounds revealed a weak but statistically significant global separation among treatments (PERMANOVA, R^2^ = 0.23, F = 1.56, *P* = 0.020; Fig. 5A). However, no individual metabolites remained significant after FDR correction (all adjusted *P* > 0.05; Table S5). Therefore, effect size analysis was integrated with RF variable importance to identify metabolites consistently associated with treatment responses. Several compounds exhibited large global and pairwise effect sizes, indicating consistent treatment-related shifts (Table S8).

Under D treatment, only a few metabolites met these criteria, namely those showing large effect sizes (η^2^[H] ≥ 0.14 and/or VDA ≥ 0.71) and statistical importance in RF models (permutation-based *P* < 0.05). Among them, sucrose and tartaric acid displayed large effect sizes and were identified as significant contributors in the RF model, both tending to exhibit lower intensities in water-limited plants compared with C (Tables S5 and S8; Fig. 5B). 5-Oxoproline emerged as a key metabolite distinguishing O from C, showing a large effect size and a tendency toward higher levels in O plants (Tables S5 and S8; Fig. 5C). In DO plants, gluconic acid and several sugars also exhibited large effect sizes, increased relative abundance, and strong discriminatory power in the RF model (Tables S5 and S8; Fig. 5D).

**Fig. 6:**
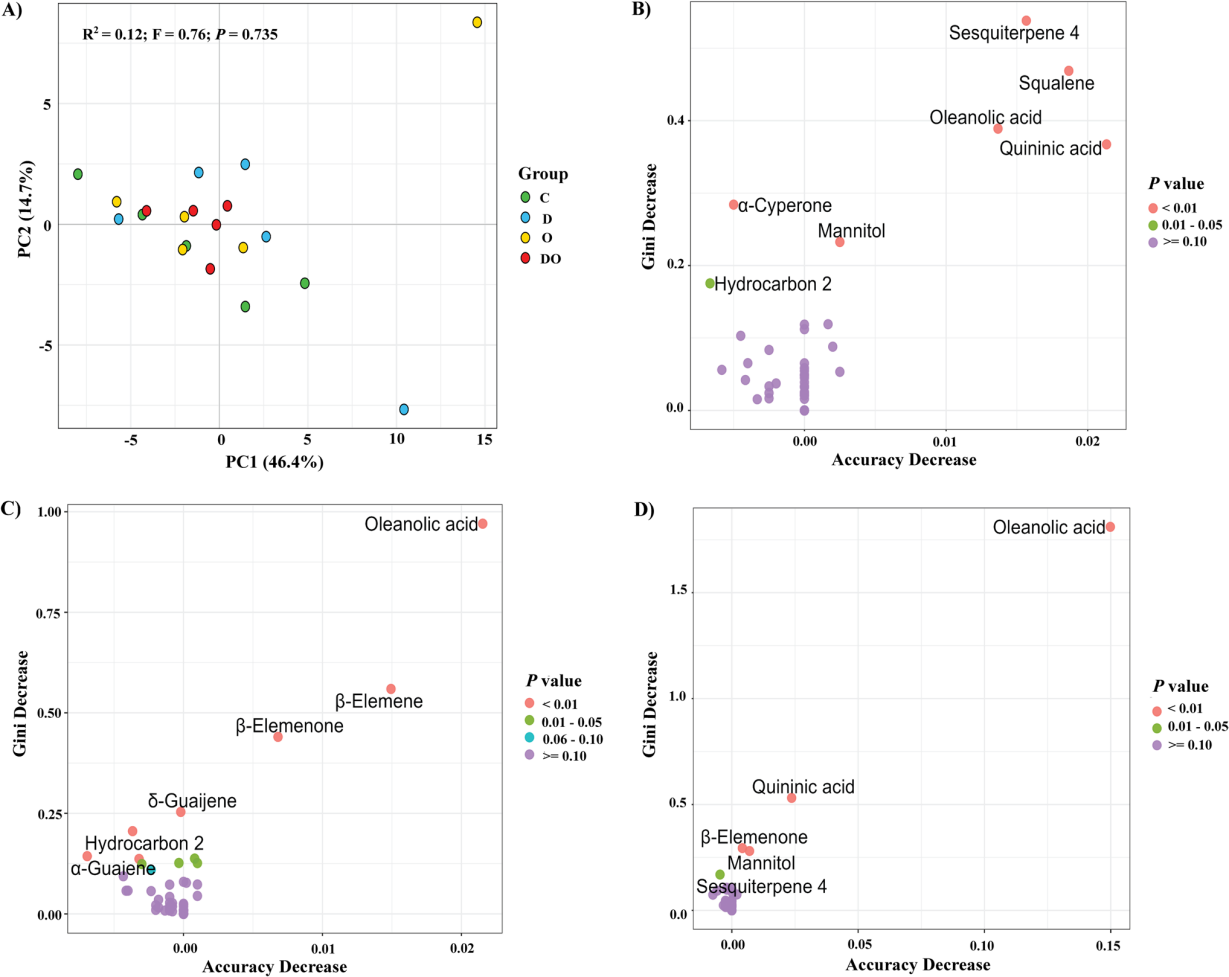
Multivariate analyses of the nonpolar leaf metabolome of *Eugenia uniflora* saplings under drought and ozone stress. **A** PCA score plot of the based on annotated metabolites. **B–D** Random Forest variable importance plots for pairwise comparisons in the polar phase: C vs D (**B**), C vs O (**C**), and C vs DO (**D**). In the Random Forest plots, colors represent permutation-derived p-value thresholds indicating the statistical relevance of each variable. *C* control (*n* = 5), *D* drought (*n* = 5), *O* ozone (*n* = 5), *DO* combined drought + ozone (*n* = 5)

For the nonpolar phase, neither multivariate nor univariate analyses revealed statistically significant treatment effects after correction (all adjusted *P* > 0.05; Fig. 6A; Table S7). Nevertheless, the triterpene oleanolic acid showed large effect sizes, with relatively moderate intensities in C and even higher values in O, emerging as one of the most influential variables in RF models (Tables S7 and S8; Fig. 6B–D). In contrast, total sesquiterpene (SQT and OSQT) levels remained relatively stable across treatments in the nonpolar metabolomic profile, despite a tendency toward reduced SQT emissions in the O group (Table 2).

## Discussion

### Effects of short-term water limitation on *Eugenia uniflora* saplings

Drought is one of the most pervasive abiotic constraints limiting plant growth and survival, and its impacts are expected to intensify under climate change (Seleiman et al. 2021). Urban ecosystems are projected to experience increasing temperatures and aridity, exacerbating water limitation. Notably, niche-based projections indicate that *E. uniflora* populations in southeastern and central Brazil already operate near their climatic safety margins, particularly regarding seasonal water availability (Esperon-Rodriguez et al. 2022). In this context, our results demonstrate that short-term water deprivation induced only mild biochemical and physiological perturbations in *E. uniflora* saplings, consistent with an early or mild acclimatory response to water limitation.

Reduced soil moisture is a major driver of plant water stress, and leaf RWC is a reliable indicator of plant water status and drought tolerance (González and González-Vilar 2001; Gupta et al. 2012). In the present study, despite significant reductions in soil moisture, leaf RWC remained stable, indicating effective maintenance of cellular hydration. This response is consistent with mechanisms associated with drought avoidance strategy, in which plants maintain tissue hydration under limited water availability (Bandurska 2022). Similar responses have been reported for drought-tolerant species under mild stress, whereas pronounced declines in RWC generally occur only under severe or prolonged water deficit (Babaei et al. 2021). Consistently, Toscano et al. (2016) reported that significant reductions in RWC in *E. uniflora* subjected to well-watered, moderate, and severe drought treatments were observed only after 28 days of stress and exclusively under severe drought conditions.

The absence of visible injury and chlorosis further indicates that the imposed water limitation did not trigger acute tissue damage, although visual symptoms may lag behind physiological impairment (Hörmann et al. 2025). Previous studies have also reported that *E. uniflora* can rapidly adjust to water limitation and remain visually unaffected for more than one month under restricted irrigation, suggesting a capacity to maintain physiological stability under water-limited conditions (Toscano et al. 2016).

Gas exchange parameters and photosynthetic pigment contents were likewise preserved under water limitation. Given that stomatal closure and pigment degradation are commonly reported early responses to water deficit (Sharma et al. 2020), the maintenance of CO₂ assimilation, stomatal conductance, and chlorophyll and carotenoid levels indicates that photochemical efficiency and structural integrity of the photosynthetic apparatus were not compromised. While previous studies in *E. uniflora* and *Psidium myrtoides* (Myrtaceae) have documented reductions in gas exchange under moderate to severe drought (Toscano et al. 2016; Souza et al. 2023), the absence of such responses in the present study suggests that the imposed water limitation did not result in a physiologically significant drought stress. Rather, the applied treatment represents a mild or early-stage water limitation, under which physiological parameters such as stomatal conductance and leaf water status may remain stable while biochemical and metabolic adjustments are initiated. This discrepancy likely reflects differences in stress intensity and duration, as well as experimental conditions such as soil characteristics. In the present experiment, saplings were maintained at field capacity until the onset of treatments, and the imposed water limitation was of mild intensity and short duration, conditions that likely limited physiological disruption.

The preservation of photosynthetic performance was paralleled by stable non-enzymatic antioxidant pools. Neither AsA nor GSH contents declined under water limitation, and redox ratios remained above 0.5, indicating sustained intracellular redox balance. Although drought-tolerant species frequently exhibit enhanced antioxidant accumulation (Sakhno et al. 2019), the absence of marked changes here suggests that oxidative pressure remained mild to moderate and that constitutive antioxidant capacity was sufficient to buffer stress-induced ROS formation.

In contrast to the overall stability of primary physiological traits, water limitation induced qualitative and multivariate shifts in BVOC emission profiles without altering total emission rates. This selective modulation suggests targeted reprogramming of volatile composition rather than generalized upregulation. The association of methyl salicylate and β-ocimene with water deprived saplings suggests the involvement of stress-related signaling and defense pathways. Methyl salicylate functions in intra- and interplant communication and responds to both biotic and abiotic stressors (Gong et al. 2023), while β-ocimene is widely recognized as a stress-responsive monoterpene induced under diverse environmental conditions, including both biotic and abiotic stresses (Schausberger et al. 2012; Li et al. 2024). Moreover, greater inter-individual variability in volatile emissions under water limitation was observed in *E. uniflora*, which may reflect increased biochemical plasticity under stress conditions. However, we acknowledge that part of this variability could also arise from differences in the degree of water limitation among individual pots due to heterogeneous soil drying. This contrasts with the more uniform responses reported in some crop species (Bell et al. 2025), highlighting that BVOC regulation under water deficit is strongly species-specific (Anselmo-Moreira et al. 2025).

Monoterpenes and sesquiterpenes were key contributors to treatment discrimination in our dataset, suggesting that compound-specific changes in these terpenoid groups contributed to the observed stress-related variation in the BVOC profile. These terpenoids contribute to oxidative buffering and membrane stabilization under abiotic stress (Shan and Jin 2025). The stability of total emissions combined with compound-specific shifts indicates that *E. uniflora* modulates volatile blends strategically, potentially contributing to defensive signaling while minimizing carbon costs associated with BVOC production and emission.

Consistent with the limited physiological perturbation observed, osmotic reprogramming in *E. uniflora* was not pronounced. Although osmolyte accumulation, including increases in proline and soluble sugars, is a common drought response (Ghosh et al. 2022), key osmoprotective metabolites did not show consistent enrichment in water-limited saplings. Previous reports of proline accumulation in this species were associated with more severe or prolonged stress (Toscano et al. 2016). The absence of strong osmolyte induction here likely reflects mild water limitation intensity rather than reduced tolerance. Similarly, the nonpolar metabolome remained largely stable under isolated water limitation, indicating preserved membrane composition and structural integrity (Henschel et al. 2024). When considered together with the stable redox balance and the absence of pronounced osmolyte accumulation, these results reinforce that *E. uniflora* maintained structural and metabolic homeostasis without requiring large-scale metabolic reprogramming.

These findings indicate that *E. uniflora* saplings maintained physiological stability under mild, short-term water limitation, without detectable impairment in the measured parameters, accompanied by targeted metabolic adjustments. Considering projections of increasing urban aridity (Esperon-Rodriguez et al. 2022), such resilience may be ecologically relevant for the persistence of this species in urban environments. However, as reported under more prolonged or severe drought regimes (Toscano et al. 2016), tolerance thresholds are likely dose- and duration-dependent.

### Effects of ozone exposure on *Eugenia uniflora* saplings

O₃ is a highly reactive atmospheric oxidant. After entering leaves primarily through stomata, it promotes ROS generation and activates redox signaling cascades that can lead to visible foliar injury and physiological impairment. The outcome depends on the effective dose and species-specific antioxidant/detoxification capacity (Vainonen and Kangasjärvi 2015). Under the O₃ exposure in this study, *E. uniflora* saplings expressed a response pattern consistent with an oxidative challenge that remained spatially restricted and did not propagate into broad physiological impairment.

The clearest O₃-linked phenotype was the occurrence of small brown spots exclusively in fumigated saplings (O and DO). This specificity is consistent with O₃-induced visible foliar injury, which is frequently described as interveinal yellow-to-dark-brown stippling that may progress to necrotic spots (Moura et al. 2022a, 2023). Despite these mild foliar symptoms, pigment contents (chlorophylls and carotenoids) and gas exchange (stomatal conductance and CO₂ assimilation) were unchanged across treatments, suggesting minimal whole-leaf impairment at the time of measurement, which may reflect partial physiological recovery following the cessation of O_3_ exposure. Given that O_3_ fumigation was discontinued two days prior to measurements and applied intermittently (5 h day⁻^1^), the observed responses may represent residual or longer-term effects rather than acute physiological impairment. Visible injury may therefore reflect spatially restricted mesophyll damage that is sufficient to produce localized symptoms but too limited to measurably affect bulk pigment pools, chamber-averaged conductance, or net photosynthesis under moderate O₃ exposure (Vainonen and Kangasjärvi 2015; Vollenweider et al. 2019).

Consistently, the clearest biochemical response to O₃ in *E. uniflora* involved non-enzymatic antioxidants. Because O₃ can rapidly perturb cellular redox homeostasis, ascorbate- and glutathione-related metrics are widely used to characterize biochemical responses to O₃ stress (Mishra et al. 2024). O₃ exposure decreased AsA and GSH, and total pools also showed downward trends (not statistically significant), whereas the redox ratios remained high (> 0.5) and did not change statistically. This pattern suggests that O₃ increased antioxidant demand and contributed to overall pool depletion, while regeneration capacity was likely sufficient to preserve the redox distribution within the AsA–GSH cycle (Foyer and Noctor 2011). This pattern has also been observed under O₃ stress in other species. For instance, in field-grown maize, elevated O₃ reduced total foliar glutathione in some hybrids without detectable changes in glutathione redox status (Choquette et al. 2020). A comparable response has been reported in tobacco, where elevated O₃ decreased leaf-tissue AsA and AsAt, while AsA/AsAt was not significantly affected (Dai et al. 2019).

O₃ exposure did not produce major quantitative BVOC responses, as total emission rates were not statistically different among treatments. Qualitatively, no BVOCs were uniquely detected in the O treatment at the time of sampling, suggesting no persistent de novo appearance of compounds under the applied O₃ conditions. It is important to note, however, that stress-induced BVOC emissions, particularly green leaf volatiles (GLVs), are typically rapid and transient, showing sharp increases immediately after stress onset followed by rapid decline due to fast metabolic turnover (Brilli et al. 2011). Despite the lack of strong quantitative responses, multivariate analyses suggested subtle changes in BVOC composition under O₃ exposure and RF identified terpenoids as the strongest discriminators of C vs O. Notably, 3-carene was detected only in C, consistent with loss or suppression under O₃. Similar O₃-driven shifts in BVOC profiles revealed by multivariate analyses have been reported in tropical tree species (Moura et al. 2022b). In addition, metabolomic data indicated higher levels of the triterpene oleanolic acid specifically under O treatment, suggesting involvement of terpenoid-related defense pathways in response to oxidative stress.

In this context, the contrasting patterns observed between SQT emissions and their accumulation in leaf tissues suggest a potential decoupling between production and release under O₃ exposure. While emissions tended to decrease, the higher abundance of SQTs and OSQTs in the nonpolar metabolome indicates that these compounds may be retained or differentially allocated within leaf tissues. One possible explanation is that a fraction of the produced SQTs does not reach the atmosphere but instead accumulates or is consumed within the leaf. Importantly, this interpretation highlights the need to consider the intercellular air space, where BVOCs may interact with incoming O₃ and potentially contribute to a chemical barrier against oxidative stress (Yu and Blande 2022). Thus, the reduced emissions, despite sustained or increased internal pools, may reflect not only retention but also possible in situ consumption within intercellular spaces. However, this compartment remains poorly characterized, particularly in tropical species such as *E. uniflora*, highlighting the need for future studies integrating BVOC emissions with estimates of intercellular BVOC concentrations.

Consistent with these patterns, GC–MS profiling indicated limited metabolomic variation associated with O₃ exposure. In the polar phase, O₃-containing treatments contributed to a modest shift in the overall metabolite profile. However, no individual compound or class remained significant after FDR correction. Still, among the metabolites showing large effect sizes and high importance in the RF analysis, 5-oxoproline tended to occur at higher levels in the O₃-containing treatments. This metabolite is involved in glutathione metabolism and has been previously associated with tolerance to abiotic stress (Lei et al. 2024). In the nonpolar phase, the overall metabolite profile showed no robust treatment separation, although oleanolic acid displayed the clearest O₃-associated trend. This compound is an oxygenated pentacyclic triterpenoid with documented antioxidant activity (including inhibition of lipid peroxidation and ROS-scavenging capacity), and its abundance can increase under oxidative abiotic stress in plants (Günther and Bednarczyk-Cwynar 2025).

Our results provide a complementary perspective on the O₃ susceptibility of *E. uniflora*. Engela et al. (2021) classified this species as O₃-sensitive after chronic exposure in an O₃-FACE system lasting 75 days (AOT40 approximately 43,881 ppb·h), which resulted in systemic effects including reduced photosynthesis, stomatal conductance, and biomass production. In our experiment, O₃ was applied as an acute short-term episode (6 days, 5 h per day), and cumulative AOT40 reached 874.6 ppb·h, indicating an integrated exposure about 50-fold lower than in the FACE study. This large difference in exposure duration and cumulative dose likely contributes to explaining why we detected localized visible lesions and redox-associated responses, without consistent changes in overall photosynthetic pigment content or net gas exchange. Acute short-term exposures can elicit spatially restricted injury and biochemical turnover, whereas chronic regimes are more likely to propagate into sustained constraints on carbon assimilation and growth (Vainonen and Kangasjärvi 2015). These contrasting results likely reflect differences in exposure duration and cumulative O₃ dose, which strongly influence the magnitude and systemic expression of O₃ effects in *E. uniflora*.

### Responses of *Eugenia uniflora* saplings to combined drought and ozone stress

The interaction between drought and O_3_ represents a complex physiological scenario in which hydraulic limitation and oxidative stress co-occur. While drought primarily constrains plant function through reduced water availability and potential stomatal closure, O_3_ imposes oxidative pressure after entering leaves via stomatal uptake and subsequently generating ROS. When combined, these stressors may interact in synergistic, additive, or antagonistic ways depending on stress intensity, duration, the order of stress imposition, and species-specific regulatory capacity (Zandalinas and Mittler 2022). Although contrasting responses have been reported, drought–O_3_ interactions often result in partially antagonistic outcomes (Suzuki et al. 2014), as water deficit–induced stomatal closure can reduce O_3_ influx and thereby mitigate its phytotoxic effects. Because O_3_ uptake occurs predominantly through stomata, drought-driven stomatal limitation may partially reduce O_3_ uptake and thereby mitigate oxidative effects (Matyssek et al. 2006).

In addition to stomatal regulation, drought–ozone interactions may also be influenced by metabolic constraints. Under more severe or prolonged water limitation, reduced carbon availability could limit the synthesis or maintenance of antioxidant defenses, thereby affecting ozone detoxification capacity (Matyssek et al. 1995; Grantz et al. 2013). Although our results did not provide direct evidence for this mechanism, they suggest that metabolic and biochemical adjustments may contribute to the outcome of combined stress even in the absence of detectable changes in stomatal conductance.

Here, the DO treatment did not result in pronounced physiological impairment relative to the individual stresses. Leaf RWC remained comparable to both the C and the single-stress treatments, while gas exchange parameters and photosynthetic pigment contents were likewise preserved. Moreover, no widespread increase in visible injury was observed beyond the localized brown spots associated with O₃ exposure. Together, these results indicate that the combined stress did not disrupt leaf water status or lead to measurable impairment of photosynthetic performance under the experimental conditions. Similar patterns have been reported in other species exposed to simultaneous drought and O_3_ stress. For example, in *Medicago truncatula*, the combined treatment did not significantly affect chlorophyll content or photosynthetic activity (Iyer et al. 2013). Likewise, in poplar plants exposed to O_3_ and water stress for three months, physiological parameters such as photosynthesis and biomass differed among treatments, but drought partially mitigated O_3_-induced damage, suggesting that water limitation may exert a protective effect against O_3_ stress (Gao et al. 2017).

As previously mentioned, although O₃ exposure alone decreased AsA and GSH, their concentrations in the DO treatment were higher than in O group, suggesting a tendency toward attenuation of O_3_-driven antioxidant depletion under concurrent water limitation. Antagonistic or mitigating interactions between drought and O_3_ affecting antioxidant responses have also been reported in other species. For instance, in *Medicago truncatula*, AsA levels were higher under combined drought and O_3_ stress than under O_3_ exposure alone (Iyer et al. 2013).

This pattern suggests that the attenuation of O₃-induced oxidative stress under combined conditions may not be restricted to the AsA–GSH cycle alone. Although the present study focused on this pathway, other non-enzymatic antioxidant systems may also contribute to oxidative stress mitigation. Phenolic compounds, for example, represent an important class of antioxidants involved in ROS scavenging and stress tolerance (Kumar et al. 2023). Supporting this broader perspective, seedlings of *Quercus robur* exposed to combined drought and O₃ stress exhibited higher concentrations of total phenolics compared with O₃ alone, while drought-treated plants showed phenolic levels similar to those observed under combined stress, suggesting a potential antagonistic interaction between the two stressors (Peron et al. 2021).

As observed for the O treatment, combined stress in *E. uniflora* did not result in quantitative differences in total BVOC emissions, and no BVOCs were uniquely induced under the DO treatment. However, multivariate analyses revealed clear compositional shifts in BVOC profiles under combined stress. This shift was driven mainly by farnesane, which PCA and RF analyses identified as the main compound separating the DO treatment from the control. Farnesane is a sesquiterpene and the saturated analogue of farnesene, which have been associated with abiotic stress responses and defense signaling (Palmer-Young et al. 2015). Although the specific role of farnesane remains unclear, its prominence in the DO treatment further supports that sesquiterpenes may be associated with stress-induced shifts in BVOC composition under combined stress in *E. uniflora*.

Similar patterns have been reported in other species exposed to concurrent drought and O₃ stress. For instance, in *Brassica nigra*, total BVOC emissions were not significantly affected by O₃ or drought applied individually or in combination, although the BVOC blend differed among treatments (Kask et al. 2021). Likewise, in *Phillyrea angustifolia*, terpene emissions remained relatively stable under drought and O₃ exposure, with no significant interaction between watering regime and O₃ (Pellegrini et al. 2021).

At the metabolomic level, the DO treatment induced subtle yet coordinated adjustments rather than large-scale metabolic disruption. In the polar phase, DO treatment generally showed a tendency toward higher total sugar content, with several sugars exhibiting large effect sizes. Similar patterns have been reported in other species exposed to the same stress combination. For instance, *Quercus cerris* seedlings subjected to simultaneous drought and O_3_ exposure showed significantly higher hexose concentrations compared with single-stress treatments (Cotrozzi et al. 2017). Soluble sugars perform multiple functions in plants, including roles in osmoprotection, carbon allocation, and stress signaling (Saddhe et al. 2021). The tendency toward higher sugar levels under combined stress suggests a possible contribution of osmotic adjustment to stress responses. However, given that similar responses are commonly associated with drought alone, this pattern likely reflects a general response to water limitation rather than a mechanism exclusive to the DO treatment.

In the nonpolar phase, the combined stress did not produce pronounced metabolic changes and metabolites associated with membrane structure remained relatively stable. Comparable patterns have been reported in other species, including *Vigna unguiculata* (cv. EPACE-1), where membrane lipid composition remained largely unaffected under combined stress (Rebouças et al. 2017), despite differences in stress regimes and experimental conditions.

Therefore, under the stress levels applied in this study, the interaction between drought and O₃ in *E. uniflora* saplings appeared to be partially antagonistic rather than additive or synergistic, with water limitation potentially contributing to the observed attenuation of O_3_-related effects. This response is consistent with fine-scale foliar metabolic tuning rather than compounded biochemical disruption under short-term and moderate stress conditions. This outcome likely reflects the short-term and relatively mild nature of applied stress, which may have limited the emergence of synergistic effects initially hypothesized. Under these conditions, plant responses appeared to prioritize physiological stability while still allowing measurable metabolic adjustments.

Our results do not support the initial expectation of a rapid synergistic amplification of physiological responses under combined stress. However, they provide partial support for the hypothesis that water limitation-induced physiological adjustments can modulate plant responses to O_3_ exposure. Although no significant changes in stomatal conductance were observed, the interaction between water limitation and O_3_ exposure resulted in a distinct response pattern, including the mitigation of antioxidant depletion and the absence of major physiological impairment. This suggests that mechanisms beyond stomatal regulation, such as metabolic and biochemical adjustments, may contribute to the modulation of O_3_ effects under combined conditions.

In addition, our findings indicate that internally accumulated BVOCs may contribute to plant defense responses under stress conditions. The production of distinct BVOCs across treatments suggests a treatment-specific metabolic reprogramming toward defense, while the prominence of farnesane under combined stress suggests that specific C15 volatile hydrocarbons may be associated with stress-induced shifts in BVOC composition. Together, these results indicate that *E. uniflora* exhibits a regulated response pattern to combined stress conditions, indicating that water limitation alters O_3_ effects through coordinated physiological and metabolic adjustments.

We acknowledge that O₃ exposure was applied at the chamber level, and thus chamber and O₃ effects cannot be fully disentangled. The two chambers were identical in construction and were operated within the same laboratory under the same general experimental schedule; however, potential chamber effects cannot be entirely excluded. While chamber effects are a recognized limitation in controlled fumigation experiments, particularly when a single chamber is used per treatment level (e.g., control vs. O₃ exposure) (Pellegrini et al. 2021; Moura et al. 2022a), the inclusion of both well-watered and drought-treated plants within each chamber allowed us to independently assess the effects of water limitation. Therefore, our results should be interpreted with caution regarding O₃-specific effects, while still providing meaningful insights into physiological and metabolic responses under combined stress conditions. Future studies including repeated experimental runs and experimental designs incorporating chamber alternation between treatments will be important to confirm the consistency of the observed responses and to further reduce potential chamber-related biases (Cardoso-Gustavson et al. 2014).

## Conclusions

*Eugenia uniflora* saplings exhibited a high degree of stability under the short-term and mild water limitation and O₃ regimes applied in this study, maintaining leaf water status, gas exchange, and photosynthetic pigment contents despite localized O₃ injury and subtle biochemical adjustments. O_3_ exposure alone imposed a clearer oxidative constraint, reflected in the depletion of reduced ascorbate and glutathione, whereas water limitation induced only mild physiological disturbance accompanied by selective shifts in BVOC composition and limited metabolomic reorganization. In addition, our findings suggest that BVOCs may play a role not only as emitted signals but also as internal components associated with plant defense responses under stress conditions. The stability of sesquiterpene pools, together with the production of treatment-specific compounds, indicates a reprogramming of terpenoid metabolism according to the type of environmental condition imposed. The prominence of farnesane under combined stress further suggests that specific C15 volatile hydrocarbons may be associated with BVOC profile adjustment under combined ozone and drought stress. Overall, *E. uniflora* exhibits coordinated physiological stability and targeted metabolic adjustments under the short-term and mild stress conditions applied in this study. Contrary to our initial hypothesis, the combined treatment did not result in a synergistic amplification of physiological responses but instead was characterized by the maintenance of physiological function accompanied by subtle yet consistent metabolic changes. These findings suggest that, under these conditions, responses to the combined treatment were primarily expressed at the metabolic level, while more pronounced physiological effects may require longer exposure periods or higher stress intensity.

These findings are particularly relevant for urban and restoration contexts, where mild-to-moderate water limitation and air pollution frequently co-occur. Future studies exploring longer exposure periods, different stress intensities, and direct measurements of stomatal O₃ flux, as well as integrating BVOC emissions with internal and intercellular metabolite dynamics, will be essential to further elucidate the mechanisms underlying these interactions.

## Supplementary Information

Below is the link to the electronic supplementary material.

Supplementary file1 (DOCX 4626 KB)

## Data Availability

The polar and non-polar phase GC–MS data (.cdf files) have been deposited in the MassIVE repository (https://massive.ucsd.edu) under accession number MSV000101240. Links to the GNPS workflows and job results are provided in the Supplementary Information. Additional raw and processed data are available from the corresponding author upon reasonable request.

## Abbreviations

AOT40: Accumulated ozone exposure over a threshold of 40 ppb
AsA: Reduced ascorbic acid
AsAt: Total ascorbic acid
BVOCs: Biogenic volatile organic compounds
GSH: Reduced glutathione
GSHt: Total glutathione
OSQTs: Oxygenated sesquiterpenes
RF: Random forest classification model
RWC: Relative water content
SQTs: Non-oxygenated sesquiterpenes
