# Supplementary material for: Drought modulates ozone stress through BVOCs, antioxidant defenses, and metabolic responses in a tropical tree
Source: Planta. 2026 Jul 20;264(3):63. doi: 10.1007/s00425-026-05033-8 (PMC13384984; doi:10.1007/s00425-026-05033-8)
Supplement: Supplementary file 1 — Supplementary file1 (DOCX 4626 KB) [file 425_2026_5033_MOESM1_ESM.docx]

**Drought modulates ozone stress through BVOCs, antioxidant defenses, and metabolic responses in a tropical tree**

Journal: Planta

Fernanda Anselmo-Moreira, Alice Claude, Alex do Nascimento, Bruno Ruiz Brandão da Costa, Ivan Hurtado-Caceres, Manon Rocco, Michael Staudt, Adalgiza Fornaro, Agnès Borbon, Cláudia Maria Furlan, Silvia Ribeiro de Souza

Corresponding authors:

^1^Fernanda Anselmo-Moreira (email: fernanda.anselmo@alumni.us.br; phone number: +5511-50676159)

^1^ Silvia Ribeiro de Souza (email: sribeiro@sp.gov.br; phone number: +5511-50676159)

^1^ Departamento de Uso Sustentável de Recursos Naturais, Unidade Jardim Botânico, Instituto de Pesquisas Ambientais, São Paulo, SP, 04301-002, Brazil.


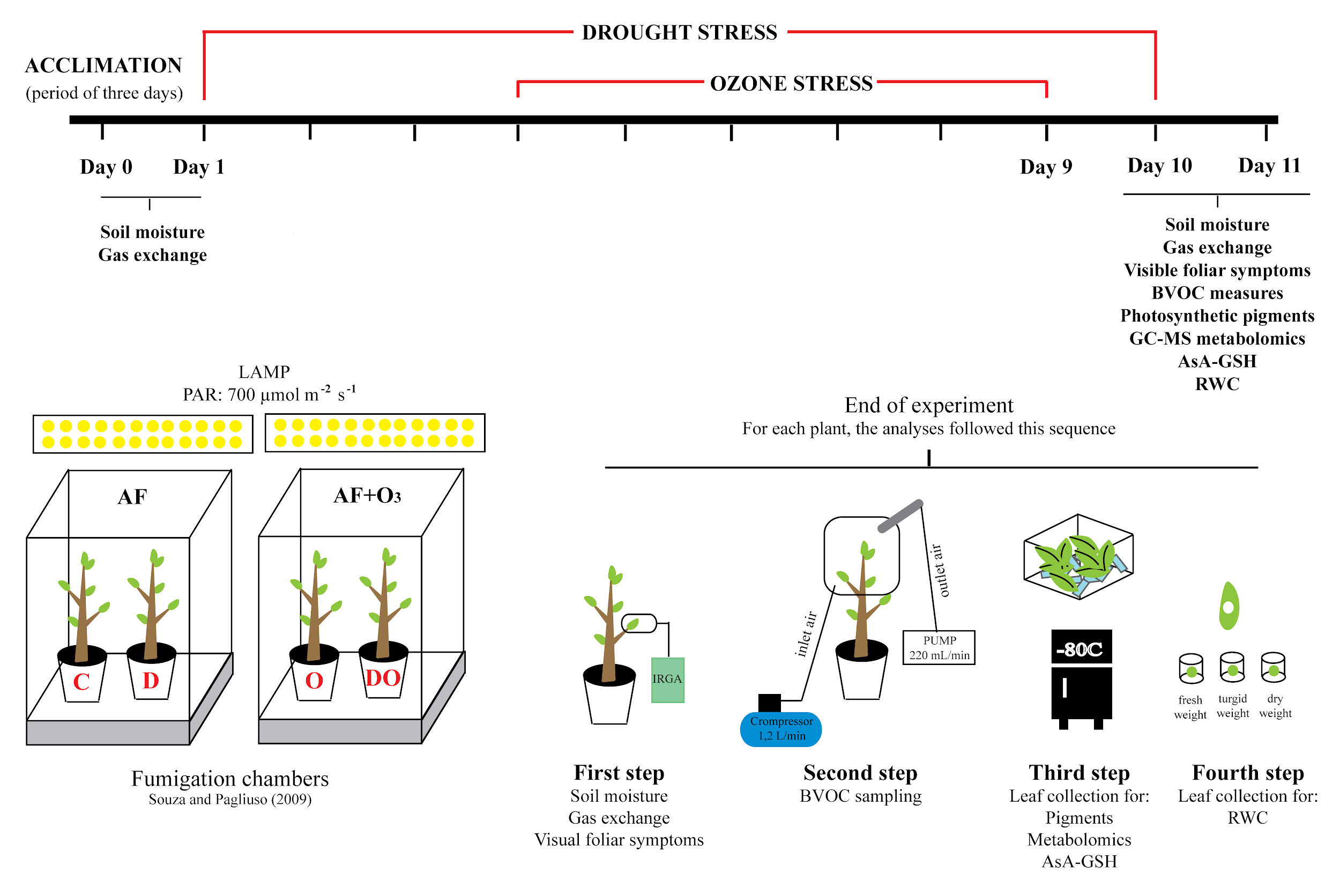


**Fig. S1** Schematic representation of experimental design. *Eugenia uniflora* seedlings were subjected to control (C), drought (D), ozone (O), and combined stress (DO) treatments over 10-days. The first three days (Day 0) correspond to the acclimation phase. Drought was applied from Day 1 to Day 10, and ozone fumigation was performed from Day 4 to Day 9. AF: air filtered. AF+O_3_: air filtered plus ozone (80-120 ppb)


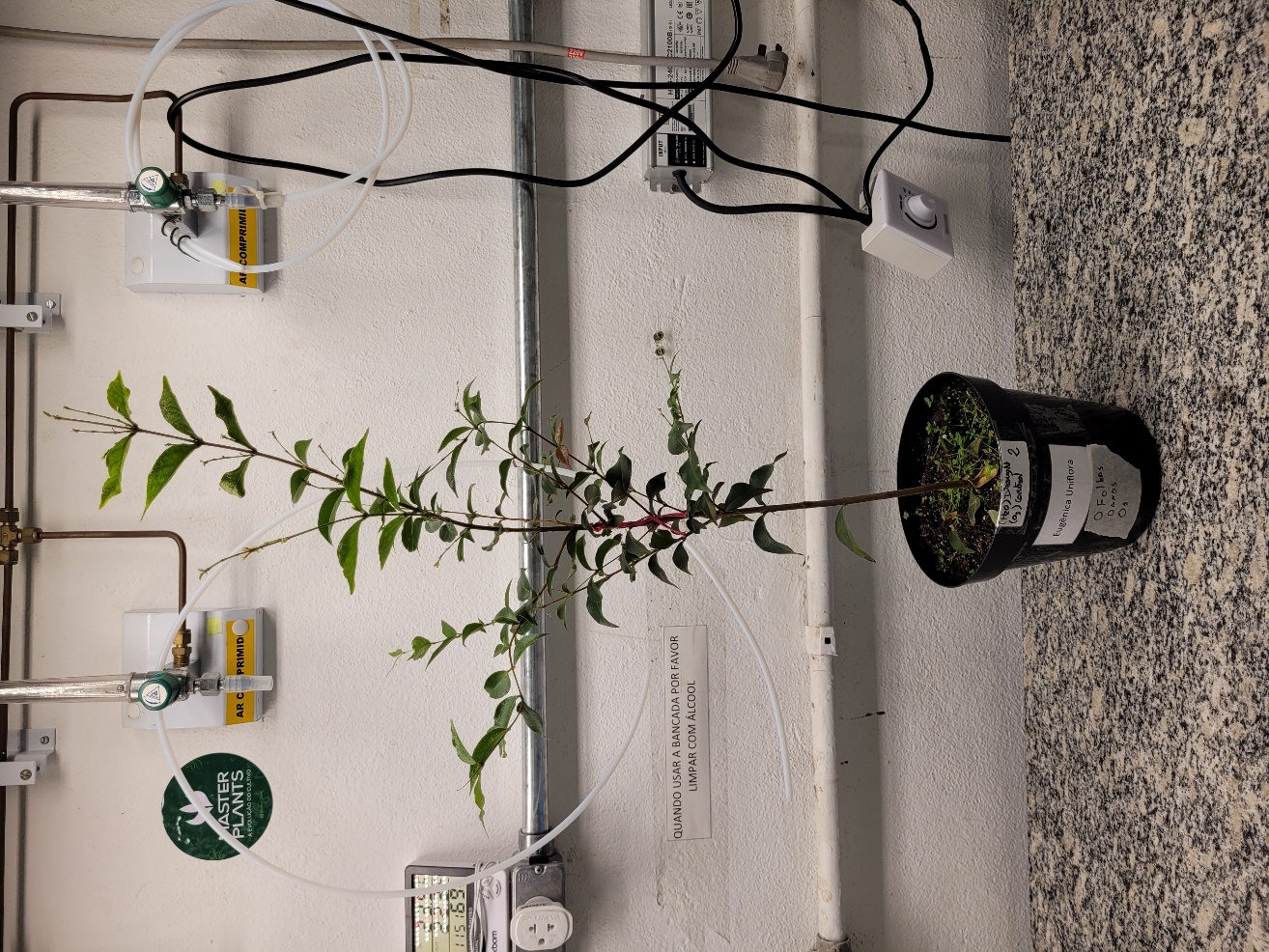


**Fig. S2** Representative *Eugenia uniflora* seedling used in the experiment, illustrating plant size and architecture at the beginning of the experiment


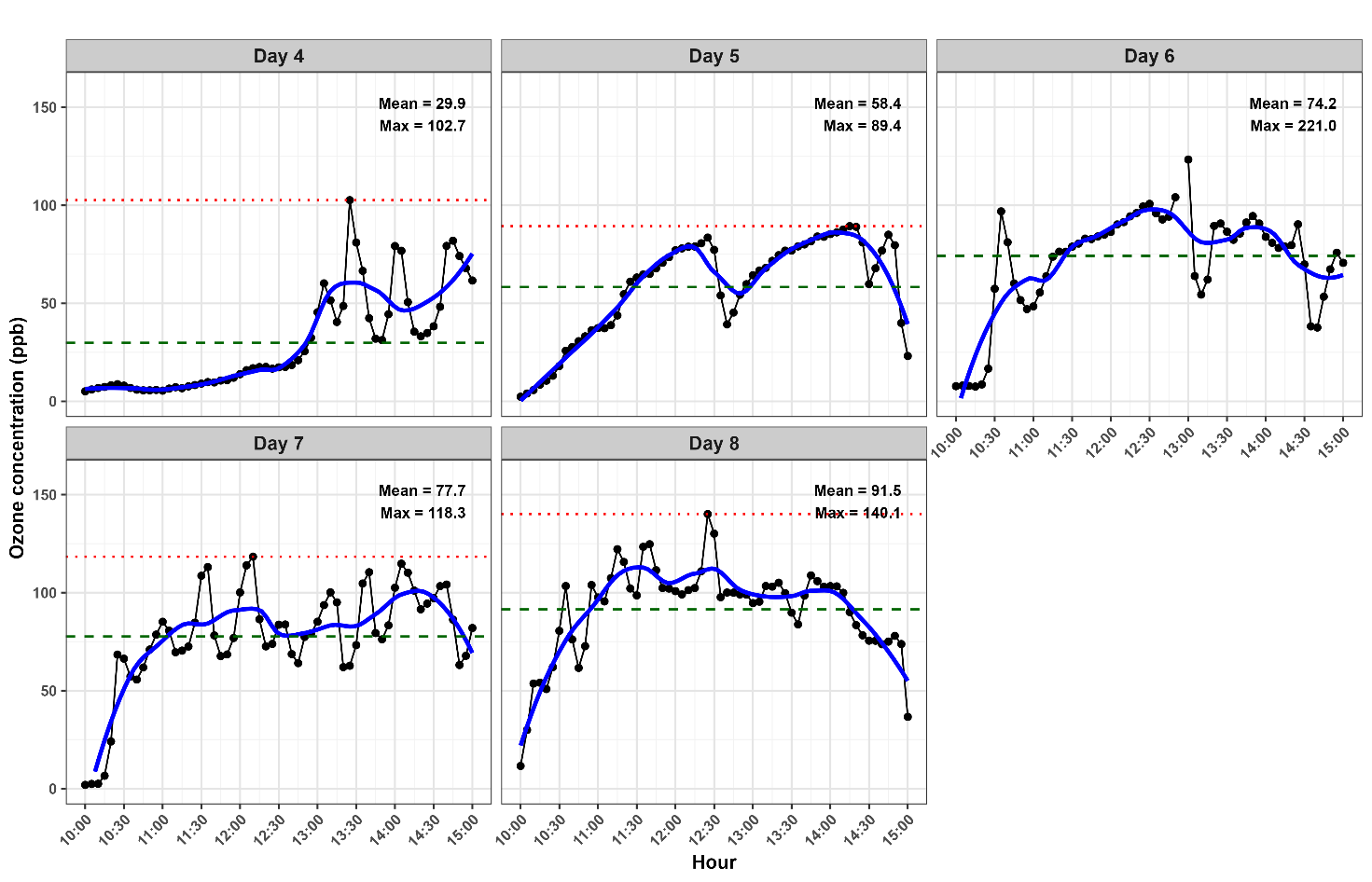


**Fig. S3** Instantaneous O₃ concentrations recorded at 5-min intervals during ozone fumigation from 10:00 to 15:00 h. Each panel represents one fumigation day with available 5-min records. Black points and lines indicate recorded O₃ concentrations, the blue line represents the smoothed temporal trend, the green dashed line indicates the daily mean O₃ concentration, and the red dotted line indicates the maximum recorded O₃ concentration for each day. For the final fumigation day, 5-min O₃ concentration records were not available due to a technical data-recording issue


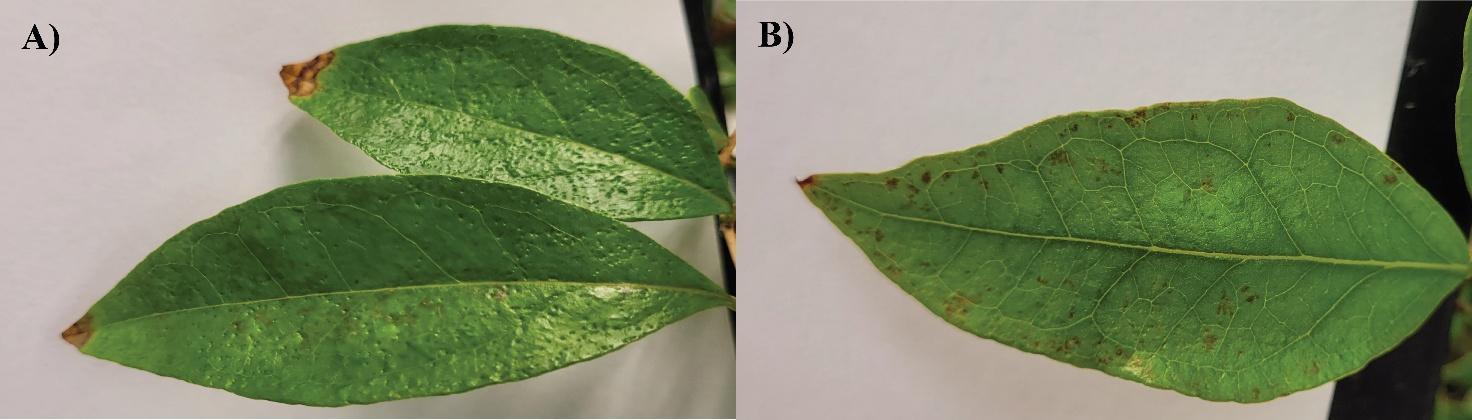


**Fig. S4** Visual leaf symptoms in *Eugenia uniflora* seedlings exposed to drought and ozone stresses. **A** Dry leaf tips. **B** Small brown spots


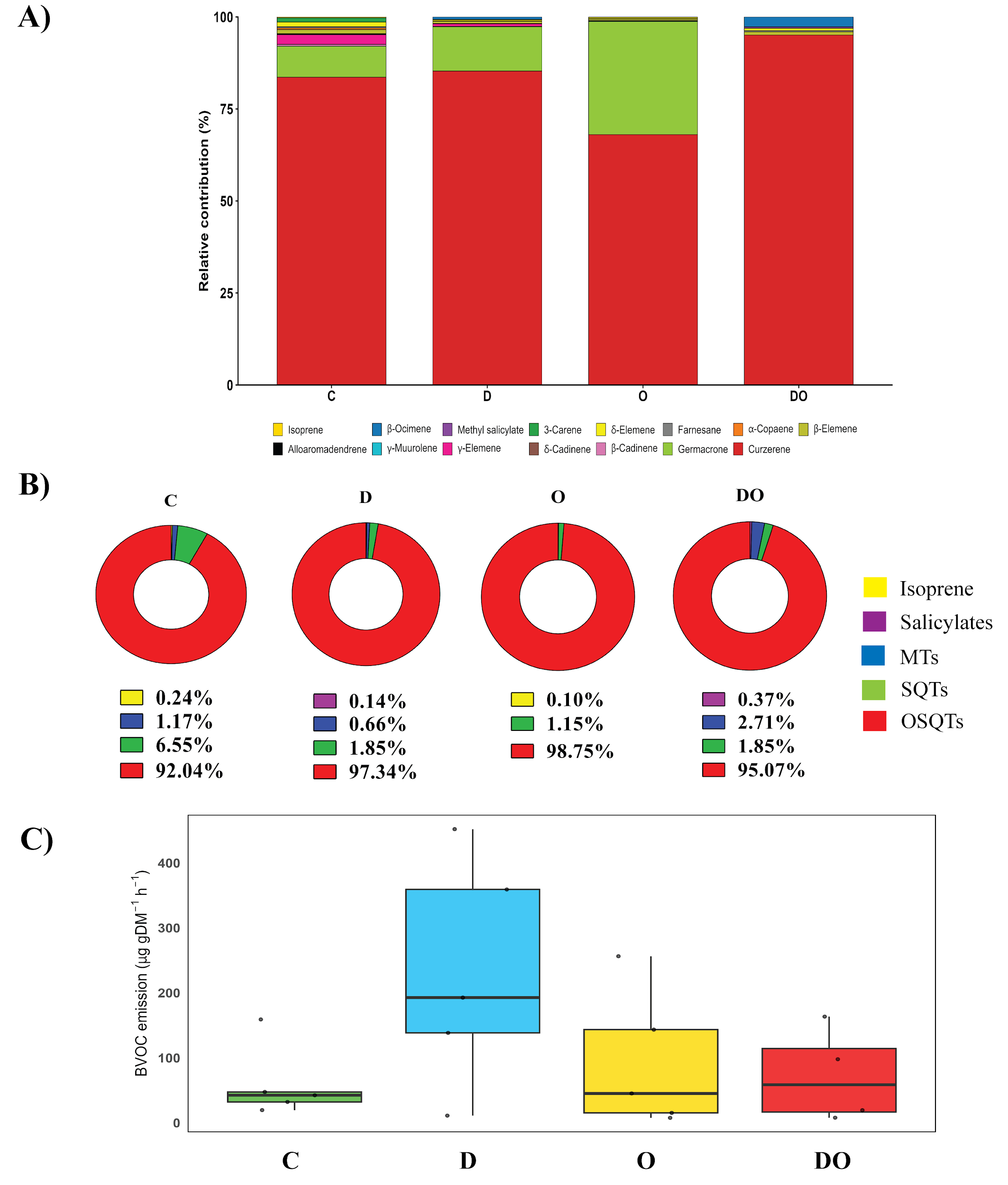


**Fig. S5** Overview of biogenic volatile organic compound (BVOC) emission profiles in *Eugenia uniflora* seedlings under different treatments. **A** Stacked percentage bar plot illustrating the relative contribution of individual BVOCs to the total emission profile of each treatment. **B** Donut plots showing the relative contribution (%) of BVOC chemical classes across treatments. **C** Total BVOC emissions (µg g⁻¹ DM h⁻¹) in *Eugenia uniflora* seedlings. C (control), D (drought), O (ozone), and DO (combined drought + ozone). MTs: non-oxygenated monoterpenes, SQTs: non-oxygenated sesquiterpenes, and OSQTs: oxygenated monoterpenes


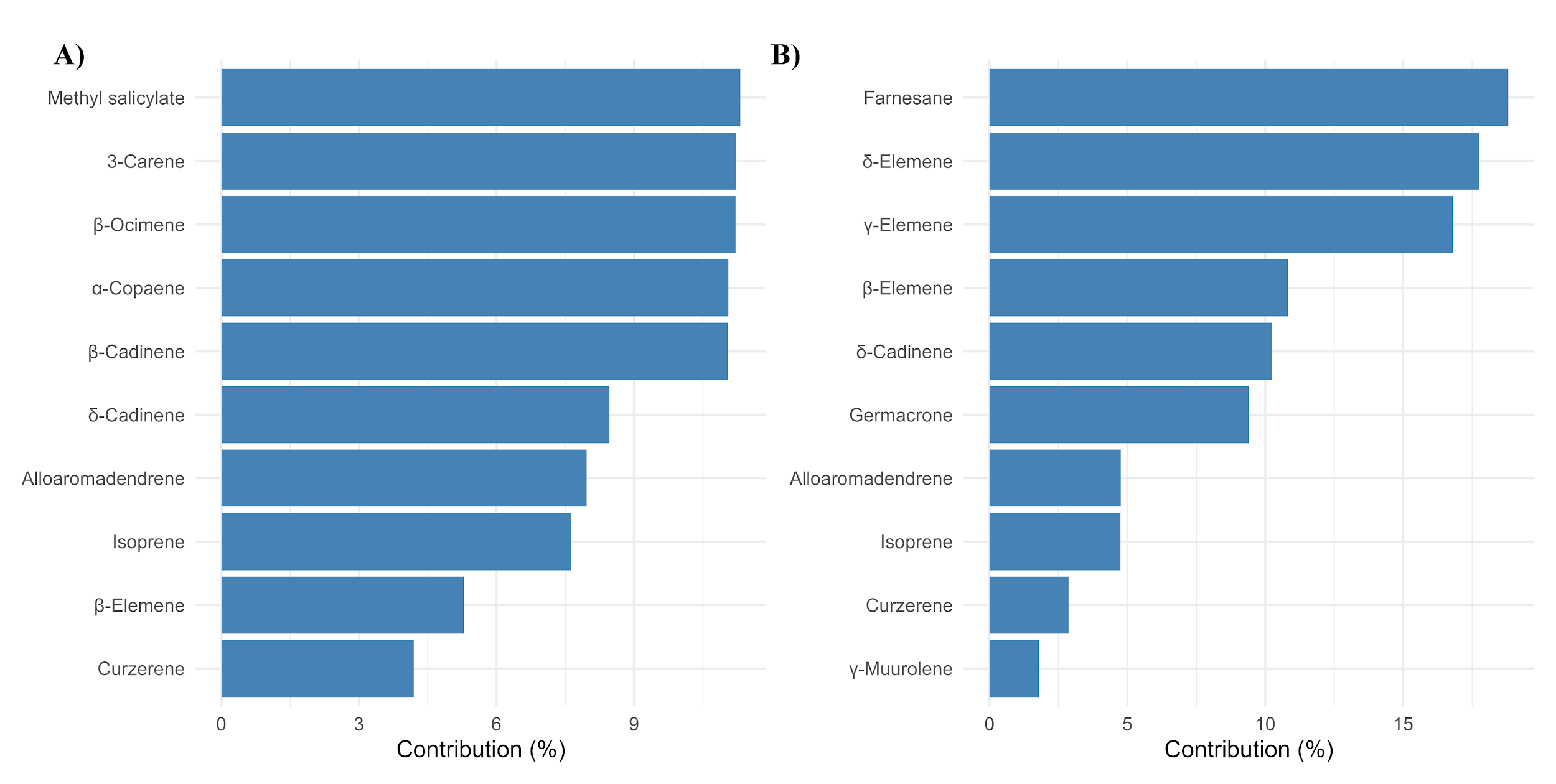


**Fig. S6** Key BVOCs driving separation along the first two principal components in the PCA analysis. **A** Top ten compounds contributing to PC1, expressed as relative contribution (%) to the variance explained by this axis. **B** Top ten compounds contributing to PC2


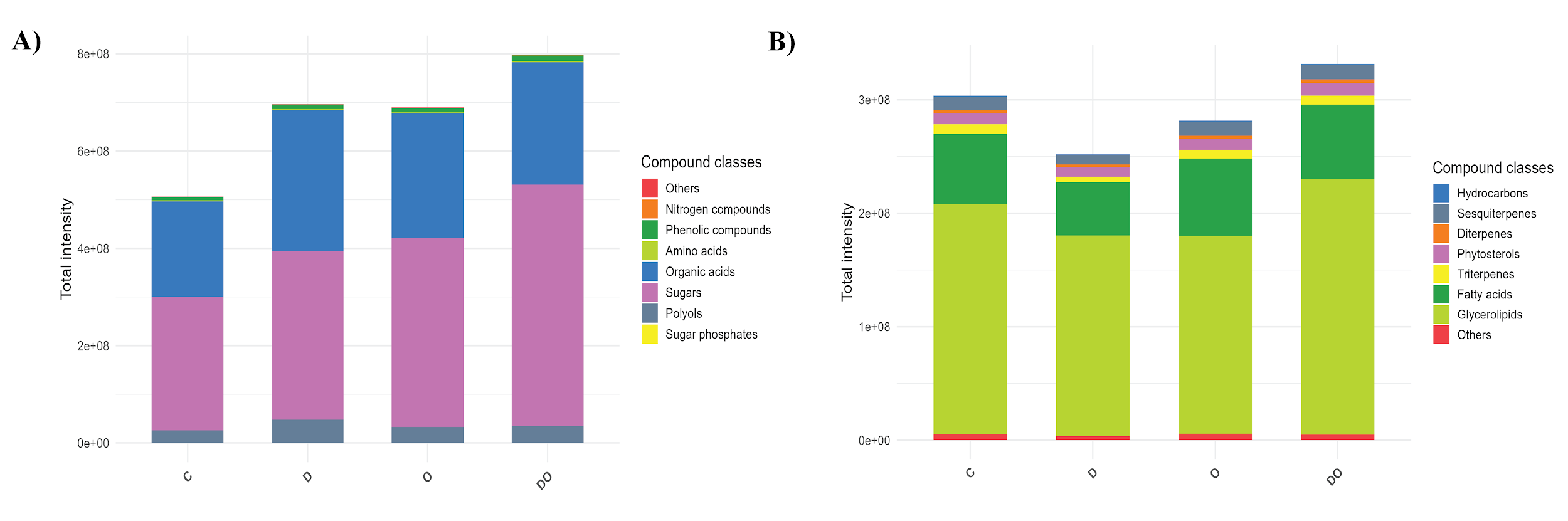


**Fig. S7** Absolute abundance of chemical classes detected by GC-MS in *Eugenia uniflora* leaf extracts. **A** Polar phase. **B** Nonpolar phase. Bars represent the summed intensities of identified classes across treatments. C: control; D: drought; O: ozone; DO: combined drought + ozone

**Table S1** Daily ozone exposure during the fumigation period

| **Fumigation day** | **Mean O_3_ (ppb)** | **Maximum O_3_ (ppb)** | **AOT40 (ppb h)** |
| --- | --- | --- | --- |
| Day 4 | 29.0 | 102.7 | 33.6 |
| Day 5 | 56.5 | 89.4 | 115.6 |
| Day 6 | 72.1 | 221 | 175.6 |
| Day 7 | 75.2 | 118.3 | 186.3 |
| Day 8 | 88.7 | 140.1 | 251.3 |
| Day 9 | – | – | 168.2 |
| **Total** |  |  | **930.7** |

Daily mean O₃ concentration, maximum O₃ concentration, and accumulated AOT40 values recorded during ozone fumigation. Fumigation was performed daily from 10:00 to 15:00 h. AOT40 represents the accumulated ozone exposure above a threshold of 40 ppb during the fumigation period and is expressed as ppb h. The dash (–) indicates that instantaneous O₃ concentration data were not available because the 5-min records were corrupted due to a technical data-recording issue; however, the cumulative AOT40 value for that day was retained and recorded during the experiment.

**Table S2** Gas exchange measurements in *Eugenia uniflora* seedlings exposed to drought and ozone stresses, alone or in combination, comparing the beginning and the end of the experiment within each treatment

| Parameter | Group | Initial | Final | t | *P* value |
| --- | --- | --- | --- | --- | --- |
| *g*_sw_ | C | 0.034 ± 0.026 | 0.039 ± 0.021 | -0.31 | 0.773 |
|  | D | 0.043 ± 0.017 | 0.073 ± 0.080 | -0.83 | 0.452 |
|  | O | 0.046 ± 0.032 | 0.038 ± 0.015 | 0.54 | 0.618 |
|  | DO | 0.080 ± 0.026 | 0.076 ± 0.076 | 0.26 | 0.810 |
| *A* | C | 3.00 ± 1.53 | 3.15 ± 1.78 | -0.14 | 0.896 |
|  | D | 4.30 ± 1.57 | 3.43 ± 2.06 | 0.76 | 0.491 |
|  | O | 4.19 ± 2.47 | 3.88 ± 1.11 | 0.34 | 0.751 |
|  | DO | 5.21 ± 1.60 | 4.64 ± 2.75 | 0.52 | 0.630 |

Data are presented as mean ± standard deviation (*n* = 5). P-values were obtained using paired t-tests comparing the beginning and the end of the experiment within each treatment. Different letters indicate significant differences among treatments (*P* < 0.05). Absence of letters denotes non-significant differences (*P* > 0.05). Bold values indicate statistical significance at *P* ≤ 0.05. Stomatal conductance (*g_sw_*, mol m⁻² s⁻¹) and CO₂ assimilation rate (*A*, µmol CO₂ m⁻² s⁻¹). C: control; D: drought; O: ozone; DO: drought + ozone.

**Table S3** Summary of statistical tests applied to non-enzymatic antioxidant variables in *Eugenia uniflora* seedlings exposed to drought and ozone stress, alone or in combination

| **Parameter** | **Shapiro-Wilk** | | | | **Kruskal-Wallis**  ***P* value** | **Pairwise comparisons** | **Dunn test**  ***P* value** |
| --- | --- | --- | --- | --- | --- | --- | --- |
|  | **C** | **D** | **O** | **DO** |  |  |  |
| AsA | 0.179 | 0.882 | 0.312 | 0.387 | **0.023** | C x D | 0.853 |
|  |  |  |  |  |  | C x O | **0.035** |
|  |  |  |  |  |  | C x DO | 0.853 |
|  |  |  |  |  |  | D x O | **0.040** |
|  |  |  |  |  |  | D x DO | 0.853 |
|  |  |  |  |  |  | DO x O | 0.064 |
| AsAt | 0.751 | 0.912 | 0.066 | 0.108 | 0.107 | – | – |
| AsA/AsAt ratio | 0.455 | 0.093 | 0.077 | 0.685 | 0.393 | – | – |
| GSH | 0.623 | 0.271 | 0.060 | **0.016** | **0.009** | C x D | 0.468 |
|  |  |  |  |  |  | C x O | **0.012** |
|  |  |  |  |  |  | C x DO | 0.843 |
|  |  |  |  |  |  | D x O | 0.075 |
|  |  |  |  |  |  | D x DO | 0.540 |
|  |  |  |  |  |  | DO x O | **0.020** |
| GSHt | 0.379 | 0.107 | **0.042** | 0.934 | 0.068 | – | – |
| GSH/GSHt ratio | 0.458 | 0.667 | 0.904 | 0.251 | 0.245 | – | – |

AsA: reduced ascorbic acid. AsAt: total ascorbic acid. GSH: reduced glutathione. GSHt: total glutathione. C: control. D: drought. O: ozone. DO: drought + ozone. Differences among groups were evaluated using the Kruskal–Wallis test, followed by Dunn’s post hoc test with Benjamini–Hochberg (BH) correction for multiple comparisons. Significant differences were considered at *P* < 0.05 (adjusted *P* values).

**Table S4** Overview of metabolites detected in the polar phase of leaf extracts from *Eugenia uniflora* seedlings analyzed by gas chromatography coupled to mass spectrometry (GC-MS)

| Peak | Putative annotation | Class | RT  (min) | Annotation level | Cosine | LRI  (exp.) | LRI  (ref.) |
| --- | --- | --- | --- | --- | --- | --- | --- |
| 1 | Malonic acid | Organic acids | 13.67 | 2 | 0.72 | 1245 | 1221 |
| 2 | Ethanolamine | Nitrogen compounds | 14.43 | 2 | 0.81 | 1271 | 1269 |
| 3 | Aminoacid 1 | Amino acids | 14.66 | 3 | - | 1279 | - |
| 4 | Phosphoric acid | Others | 14.83 | 1 | 0.86 | 1285 | 1286 |
| 5 | Glycerol | Polyols | 14.91 | 2 | 0.98 | 1288 | 1289 |
| 6 | Proline | Amino acids | 15.24 | 1 | 0.79 | 1299 | 1294 |
| 7 | Succinic acid | Organic acids | 15.78 | 2 | 0.91 | 1320 | 1321 |
| 8 | Glyceric acid | Organic acids | 16.49 | 2 | - | 1347 | 1347 |
| 9 | Serine | Amino acids | 17.28 | 2 | 0.79 | 1378 | 1380 |
| 10 | Methylmaleic acid | Organic acids | 17.44 | 2 | - | 1384 | 1386 |
| 11 | Threonine* | Amino acids | 18.00 | 2 | - | 1406 | 1408 |
| 12 | Citramalic acid | Organic acids | 20.10 | 2 | - | 1489 | 1487 |
| 13 | Malic acid | Organic acids | 20.60 | 1 | 0.96 | 1510 | - |
| 14 | 5-oxo-proline | Amino acids | 21.15 | 2 | 0.87 | 1533 | 1529 |
| 15 | Aspartic acid | Amino acids | 21.34 | 1 | 0.88 | 1541 | 1541 |
| 16 | Erythritol | Polyols | 21.78 | 2 | - | 1560 | 1535 |
| 17 | Sugar 1 | Sugars | 22.12 | 3 | - | 1574 | - |
| 18 | Erythronic acid | Organic acids | 22.56 | 2 | - | 1592 | 1569 |
| 19 | Glutamic acid | Amino acids | 23.64 | 1 | 0.78 | 1640 | 1636 |
| 20 | Tartaric acid | Organic acids | 24.35 | 2 | - | 1672 | 1668 |
| 21 | Polyol 1 | Polyols | 26.46 | 3 | - | 1770 | - |
| 22 | Xylitol | Polyols | 26.63 | 2 | - | 1778 | 1748 |
| 23 | Ribonic acid | Organic acids | 26.87 | 2 | - | 1789 | 1799 |
| 24 | Shikimic acid | Organic acids | 27.95 | 1 | 0.94 | 1842 | 1843 |
| 25 | Citric acid | Organic acids | 28.15 | 1 | 0.82 | 1852 | 1853 |
| 26 | Isocitric acid | Organic acids | 28.24 | 1 | 0.85 | 1856 | 1843 |
| 27 | Quinic acid | Organic acids | 29.12 | 2 | 0.82 | 1899 | 1900 |
| 28 | Fructose^a^ | Sugars | 29.43 | 1 | 0.98 | 1915 | 1925 |
| 29 | Fructose^a^ | Sugars | 29.65 | 1 | 0.97 | 1927 | 1933 |
| 30 | Galactose | Sugars | 29.82 | 1 | 0.85 | 1936 | 1945 |
| 31 | Glucose^b^ | Sugars | 29.92 | 1 | 0.97 | 1941 | 1937 |
| 32 | Glucose^b^ | Sugars | 30.25 | 1 | 0.94 | 1958 | 1955 |
| 33 | Sorbitol | Polyols | 30.48 | 2 | - | 1970 | 1980 |
| 34 | Mannitol | Polyols | 30.67 | 1 | - | 1979 | 1975 |
| 35 | Galactitol | Polyols | 30.98 | 2 | - | 1996 | 1988 |
| 36 | Gluconic acid | Organic acids | 32.19 | 2 | - | 2061 | 2037 |
| 37 | Sugar 2 | Sugars | 32.48 | 3 | **-** | 2076 | 2058 |
| 38 | *Myo*-Inositol | Polyols | 33.43 | 2 | 0.97 | 2129 | 2129 |
| 39 | Polyol 2 | Polyols | 33.77 | 3 | - | 2148 | - |
| 40 | Sugar 3 | Sugars | 34.04 | 3 | - | 2163 | - |
| 41 | Sugar 4 | Sugars | 34.25 | 3 | - | 2175 | - |
| 42 | Sugar 5 | Sugars | 35.33 | 3 | - | 2237 | - |
| 43 | Glucose-6-phosphate* | Sugar phosphates | 37.73 | 2 | - | 2381 | 2392 |
| 44 | Sugar 6 | Sugars | 38.35 | 3 | - | 2420 | - |
| 45 | Sugar 7 | Sugars | 38.56 | 3 | - | 2433 | - |
| 46 | Sugar 8 | Sugars | 40.69 | 3 | - | 2560 | - |
| 47 | Sugar 9 | Sugars | 40.77 | 3 | - | 2564 | - |
| 48 | Sugar 10 | Sugars | 41.27 | 3 | - | 2592 | - |
| 49 | Sugar 11 | Sugars | 41.36 | 3 | - | 2597 | - |
| 50 | Sucrose | Sugars | 42.81 | 1 | 0.95 | 2706 | 2700 |
| 51 | Lactose | Sugars | 43.32 | 2 | 0.91 | 2745 | 2737 |
| 52 | Sugar 12 | Sugars | 45.23 | 3 | - | 2888 | - |
| 53 | Melibiose | Sugars | 46.73 | 1 | - | 2999 | - |
| 54 | Sugar 13 | Sugars | 47.65 | 3 | - | 3071 | - |
| 55 | Chlorogenic acid | Phenolic compounds | 49.06 | 1 | 0.78 | 3181 | - |
| 56 | Raffinose | Sugars | 52.51 | 2 | - | 3516 | 3505 |

RT: retention time. LRI: linear retention index. exp.: experimental. ref.: reference.

* Annotations were provided by the NIST (National Institute of Standards and Technology) spectral library integrated into the DataAnalysis software (Bruker), which was used to inspect and analyze the GC-MS chromatograms.

-: information not available.

^a^ Fructose stereoisomers produced by methoximation reaction.

^b^ Glucose stereoisomers produced by methoximation reaction.

**Table S5** Overview of metabolites detected in the nonpolar phase of leaf extracts from *Eugenia uniflora* seedlings analyzed by gas chromatography coupled to mass spectrometry (GC-MS)

| Peak | Putative annotation | Class | RT  (min) | Annotation level | Cosine | LRI  (exp.) | LRI  (ref.) |
| --- | --- | --- | --- | --- | --- | --- | --- |
| 1 | Phosphoric acid* | Others | 8.91 | 2 | - | - | 1286 |
| 2 | Elemene* | Sesquiterpenoids | 10.35 | 2 | 0.83 | - | 1343 |
| 3 | β-Elemene* | Sesquiterpenoids | 11.84 | 2 | 0.84 | - | 1389 |
| 4 | β-Caryophyllene | Sesquiterpenoids | 12.58 | 2 | 0.90 | 1421 | 1418 |
| 5 | γ-Elemene | Sesquiterpenoids | 12.92 | 2 | 0.86 | 1434 | 1435 |
| 6 | α-Guaiene | Sesquiterpenoids | 13.09 | 2 | 0.85 | 1441 | 1440 |
| 7 | β-Gurjunene* | Sesquiterpenoids | 14.03 | 2 | - | 1477 | 1475 |
| 8 | γ-Muurolene | Sesquiterpenoids | 14.17 | 2 | 0.85 | 1483 | 1480 |
| 9 | δ-Selinene | Sesquiterpenoids | 14.31 | 2 | 0.90 | 1488 | 1485 |
| 10 | δ-Guaijene | Sesquiterpenoids | 14.56 | 2 | 0.80 | 1497 | 1500 |
| 11 | γ-Cadinene | Sesquiterpenoids | 14.99 | 2 | 0.76 | 1515 | 1513 |
| 12 | σ-Cadinene | Sesquiterpenoids | 15.22 | 2 | 0.85 | 1525 | 1523 |
| 13 | Sesquiterpene 1 | Sesquiterpenoids | 15.51 | 3 | - | 1536 | 1538 |
| 14 | n-Pentadecane | Hydrocarbons | 15.59 | 1 | 0.71 | 1540 | - |
| 15 | Selina-3,7(11)-diene* | Sesquiterpenoids | 15.68 | 2 | - | 1544 | 1542 |
| 16 | Germacrene B | Sesquiterpenoids | 16.05 | 2 | 0.85 | 1559 | 1558 |
| 17 | Spathulenol* | Sesquiterpenoids | 16.56 | 2 | - | 1580 | 1578 |
| 18 | Globulol* | Sesquiterpenoids | 16.84 | 2 | - | 1592 | 1580 |
| 19 | β-Elemenone* | Sesquiterpenoids | 17.18 | 2 | - | 1606 | 1605 |
| 20 | β-Atlantol* | Sesquiterpenoids | 17.81 | 2 | - | 1634 | 1614 |
| 21 | α-Cyperone | Sesquiterpenoids | 19.28 | 2 | 0.78 | 1699 | 1706 |
| 22 | Sesquiterpene 2 | Sesquiterpenoids | 20.09 | 3 | - | 1737 | - |
| 23 | Germazone* | Sesquiterpenoids | 20.36 | 2 | - | 1749 | 1746 |
| 24 | Glycerol-3-phosphate* | Glycerolipids | 21.36 | 2 | - | 1796 | 1797 |
| 25 | Fatty acid 1 | Fatty acids | 21.82 | 3 | - | 1812 | - |
| 26 | Neophytadiene | Diterpenoids | 22.29 | 2 | 0.86 | 1841 | 1842 |
| 27 | Fatty acid 2 | Fatty acids | 22.87 | 3 | - | 1870 | - |
| 28 | (7Z,11Z)-Hexadecandien-1-ol | Fatty alcohols | 23.15 | 2 | 0.84 | 1884 | 1857 |
| 29 | Quininic acid* | Alkaloids | 23.46 | 2 | - | 1899 | 1863 |
| 30 | Sesquiterpene 3 | Sesquiterpenoids | 23.90 | 3 | - | 1921 | - |
| 31 | Hydrocarbon 1 | Hydrocarbons | 24.78 | 3 | - | 1966 | - |
| 32 | Mannitol* | Sugar alcohols | 25.02 | 2 | - | 1978 | 1975 |
| 33 | Sesquiterpene 4 | Sesquiterpenoids | 25.39 | 3 | - | 1998 | - |
| 34 | Sesquiterpene 5 | Sesquiterpenoids | 26.07 | 3 | - | 2034 | - |
| 35 | Palmitic acid | Fatty acids | 26.38 | 2 | 0.94 | 2051 | 2050 |
| 36 | Margaric acid* | Fatty acids | 28.18 | 2 | - | 2150 | 2137 |
| 37 | Phytol | Diterpenoids | 28.76 | 2 | 0.76 | 2183 | 2183 |
| 38 | Linoleic acid | Fatty acids | 29.35 | 2 | 0.93 | 2217 | 2214 |
| 39 | Linolenic acid | Fatty acids | 29.47 | 2 | 0.93 | 2224 | 2208 |
| 40 | Stearic acid | Fatty acids | 29.90 | 2 | 0.94 | 2249 | 2250 |
| 41 | 1-Monomyristin* | Glycerolipids | 32.65 | 2 | - | 2415 | 2418 |
| 42 | Arachidic acid* | Fatty acids | 33.15 | 2 | - | 2446 | 2447 |
| 43 | 2-Monopalmitin* | Fatty acids | 35.15 | 2 | - | 2576 | 2576 |
| 44 | 1-Monopalmitin* | Glycerolipids | 35.64 | 2 | - | 2609 | 2607 |
| 45 | Behenic acid* | Fatty acids | 36.17 | 2 | - | 2645 | 2644 |
| 46 | 2-Monostearin* | Glycerolipids | 37.95 | 2 | - | 2768 | 2775 |
| 47 | 1-Monostearin | Glycerolipids | 38.44 | 2 | 0.75 | 2802 | 2806 |
| 48 | Squalene | Triterpenoids | 38.84 | 2 | 0.86 | 2831 | 2833 |
| 49 | Lignoceric acid* | Fatty acids | 39.00 | 2 | - | 2843 | 2848 |
| 50 | Phytosterol 1 | Phytosterols | 40.61 | 3 | - | 2962 | - |
| 51 | Phytosterol 2 | Phytosterols | 41.91 | 3 | - | 3062 | - |
| 52 | Phytosterol 3 | Phytosterols | 42.36 | 3 | - | 3098 | - |
| 53 | Phytosterol 4 | Phytosterols | 42.63 | 3 | - | 3119 | - |
| 54 | α-Tocopherol* | Vitamins | 43.10 | 2 | - | 3078 | 3111 |
| 55 | Hydrocarbon 2 | Hydrocarbons | 44.87 | 3 | - | 3252 | - |
| 56 | Stigmasterol | Phytosterols | 45.57 | 2 | 0.77 | 3281 | 3286 |
| 57 | β-Sitosterol | Phytosterols | 47.39 | 2 | 0.81 | 3359 | 3349 |
| 58 | Oleanolic acid | Triterpenoids | 49.66 | 2 | 0.78 | 3628 | 3620 |
| 59 | Ursolic acid | Triterpenoids | 49.81 | 2 | 0.72 | 3654 | 3657 |
| 60 | Triterpene 1 | Triterpenoids | 50.10 | 3 | - | 3704 | - |
| 61 | Betulin | Triterpenoids | 50.48 | 2 | 0.74 | 3770 | 3761 |

RT: retention time. LRI: linear retention index. exp.: experimental. ref.: reference.

* Annotations were provided by the NIST (National Institute of Standards and Technology) spectral library integrated into the DataAnalysis software (Bruker), which was used to inspect and analyze the GC-MS chromatograms.

-: information not available.

**Table S6** Relative abundance (%) of polar phase metabolites detected by GC-MS in leaf extracts of *Eugenia uniflora* seedlings exposed to drought and ozone stresses, alone or in combination

| **Chemical family** | **Putative**  **annotation** | **Treatments** | | | |
| --- | --- | --- | --- | --- | --- |
|  |  | **C** | **D** | **O** | **DO** |
| Saccharides |  |  |  |  |  |
|  | Sugar 1 | 0.014 ± 0.006 | 0.016 ± 0.010 | 0.012 ± 0.007 | 0.013 ± 0.013 |
|  | Fructose | 0.477 ± 0.298 | 0.402 ± 0.276 | 0.358 ± 0.119 | 0.470 ± 0.357 |
|  | Galactose | 0.024 ± 0.010 | 0.010 ± 0.007 | 0.017 ± 0.018 | 0.022 ± 0.018 |
|  | Glucose | 0.505 ± 0.209 | 0.429 ± 0.210 | 0.391 ± 0.070 | 0.452 ± 0.185 |
|  | Sugar 2 | 0.027 ± 0.007 | 0.019 ± 0.002 | 0.017 ± 0.008 | 0.021 ± 0.006 |
|  | Sugar 3 | 0.034 ± 0.013 | 0.035 ± 0.004 | 0.035 ± 0.009 | 0.038 ± 0.008 |
|  | Sugar 4 | 0.028 ± 0.008 | 0.026 ± 0.006 | 0.029 ± 0.018 | 0.024 ± 0.004 |
|  | Sugar 5 | 0.097 ± 0.048 | 0.084 ± 0.050 | 0.105 ± 0.094 | 0.121 ± 0.074 |
|  | Sugar 6 | 0.029 ± 0.010 | 0.031 ± 0.011 | 0.022 ± 0.010 | 0.044 ± 0.021 |
|  | Sugar 7 | 0.252 ± 0.098 | 0.209 ± 0.095 | 0.212 ± 0.079 | 0.321 ± 0.177 |
|  | Sugar 8 | 0.196 ± 0.199 | 0.073 ± 0.105 | 0.072 ± 0.047 | 0.043 ± 0.038 |
|  | Sugar 9 | 0.099 ± 0.045 | 0.099 ± 0.037 | 0.096 ± 0.064 | 0.127 ± 0.062 |
|  | Sugar 10 | 0.017 ± 0.006 | 0.051 ± 0.105 | 0.001 ± 0.002 | 0.001 ± 0.001 |
|  | Sugar 11 | 0.230 ± 0.130 | 0.180 ± 0.106 | 0.245 ± 0.123 | 0.307 ± 0.128 |
|  | Sucrose | 42.70 ± 10.93 | 38.92 ± 10.30 | 43.08 ± 2.766 | 47.31 ± 11.49 |
|  | Lactose | 0.080 ± 0.053 | 0.143 ± 0.126 | 0.128 ± 0.077 | 0.140 ± 0.065 |
|  | Sugar 12 | 0.042 ± 0.030 | 0.046 ± 0.048 | 0.068 ± 0.049 | 0.041 ± 0.029 |
|  | Melibiose | 0.102 ± 0.099 | 0.021 ± 0.038 | 0.007 ± 0.006 | 0.033 ± 0.066 |
|  | Sugar 13 | 2.745 ± 0.297 | 3.109 ± 1.175 | 3.054 ± 1.236 | 4.117 ± 1.262 |
|  | Raffinose | 2.851 ± 0.501 | 3.385 ± 1.153 | 4.071 ± 0.991 | 4.056 ± 1.239 |
|  | **Total** | **50.55 ± 11.70** | **47.29 ± 12.29** | **52.02 ± 4.66** | **57.70 ± 12.87** |
|  |  |  |  |  |  |
| Polyols |  |  |  |  |  |
|  | Glycerol | 0.859 ± 0.132 | 0.854 ± 0.270 | 0.640 ± 0.197 | 0.747 ± 0.154 |
|  | Erythritol | 0.010 ± 0.004 | 0.008 ± 0.003 | 0.007 ± 0.006 | 0.004 ± 0.001 |
|  | Polyol 1 | 0.136 ± 0.077 | 0.071 ± 0.073 | 0.043 ± 0.032 | 0.083 ± 0.079 |
|  | Xylitol | 1.051 ± 0.603 | 0.690 ± 0.219 | 0.677 ± 0.435 | 0.795 ± 0.310 |
|  | Sorbitol | 0.029 ± 0.027 | 0.036 ± 0.022 | 0.039 ± 0.024 | 0.043 ± 0.030 |
|  | Mannitol | 0.307 ± 0.189 | 2.173 ± 4.600 | 0.230 ± 0.144 | 0.107 ± 0.038 |
|  | Galactitol | 0.018 ± 0.018 | 0.006 ± 0.005 | 0.007 ± 0.007 | 0.007 ± 0.004 |
|  | *Myo*-Inositol | 2.244 ± 0.447 | 2.142 ± 0.845 | 2.939 ± 1.990 | 2.268 ± 0.577 |
|  | Polyols 2 | 0.016 ± 0.003 | 0.021 ± 0.015 | 0.020 ± 0.009 | 0.026 ± 0.022 |
|  | **Total** | **4.669 ± 0.748** | **6.002 ± 4.121** | **4.603 ± 2.713** | **4.081 ± 0.837** |
|  |  |  |  |  |  |
| Sugar phosphates |  |  |  |  |  |
|  | Glucose-6-phosphate | 0.012 ± 0.001 | 0.008 ± 0.004 | 0.009 ± 0.004 | 0.003 ± 0.003 |
|  |  |  |  |  |  |
| Amino acids |  |  |  |  |  |
|  | Amino acid 1 | 0.013 ± 0.007 | 0.007 ± 0.003 | 0.009 ± 0.002 | 0.010 ± 0.003 |
|  | Proline | 0.008 ± 0.002 | 0.004 ± 0.002 | 0.004 ± 0.001 | 0.004 ± 0.001 |
|  | Serine | 0.052 ± 0.002 | 0.044 ± 0.014 | 0.045 ± 0.015 | 0.045 ± 0.016 |
|  | Threonine | 0.021 ± 0.005 | 0.016 ± 0.003 | 0.016 ± 0.004 | 0.017 ± 0.004 |
|  | 5-oxoproline | 0.073 ± 0.010 | 0.065 ± 0.032 | 0.085 ± 0.029 | 0.085 ± 0.029 |
|  | Aspartic acid | 0.086 ± 0.039 | 0.042 ± 0.016 | 0.038 ± 0.028 | 0.033 ± 0.013 |
|  | Glutamic acid | 0.323 ± 0.123 | 0.191 ± 0.056 | 0.157 ± 0.060 | 0.172 ± 0.040 |
|  | **Total** | **0.575 ± 0.179** | **0.369 ± 0.103** | **0.353 ± 0.125** | **0.364 ± 0.093** |
|  |  |  |  |  |  |
| Nitrogen compounds |  |  |  |  |  |
|  | Ethanolamine | 0.004 ± 0.001 | 0.003 ± 0.001 | 0.003 ± 0.001 | 0.003 ± 0.001 |
|  |  |  |  |  |  |
| Organic acids |  |  |  |  |  |
|  | Malonic acid | 0.016 ± 0.003 | 0.014 ± 0.013 | 0.009 ± 0.002 | 0.012 ± 0.011 |
|  | Succinic acid | 0.059 ± 0.006 | 0.052 ± 0.023 | 0.046 ± 0.021 | 0.052 ± 0.022 |
|  | Glyceric acid | 0.171 ± 0.049 | 0.164 ± 0.074 | 0.089 ± 0.039 | 0.129 ± 0.072 |
|  | Methylmaleic acid | 0.029 ± 0.003 | 0.027 ± 0.007 | 0.026 ± 0.010 | 0.024 ± 0.006 |
|  | Citramalic acid | 0.007 ± 0.001 | 0.005 ± 0.004 | 0.005 ± 0.003 | 0.006 ± 0.001 |
|  | Malic acid | 0.928 ± 0.368 | 0.802 ± 0.234 | 0.725 ± 0.244 | 0.852 ± 0.274 |
|  | Erythronic acid | 0.064 ± 0.011 | 0.060 ± 0.014 | 0.056 ± 0.028 | 0.061 ± 0.024 |
|  | Tartaric acid | 0.247 ± 0.077 | 0.236 ± 0.042 | 0.276 ± 0.085 | 0.225 ± 0.047 |
|  | Ribonic acid | 0.033 ± 0.004 | 0.032 ± 0.011 | 0.025 ± 0.010 | 0.032 ± 0.014 |
|  | Shikimic acid | 0.049 ± 0.013 | 0.044 ± 0.017 | 0.036 ± 0.029 | 0.067 ± 0.087 |
|  | Citric acid | 1.891 ± 0.465 | 1.327 ± 1.103 | 1.139 ± 0.538 | 1.424 ± 0.709 |
|  | Isocitric acid | 0.024 ± 0.008 | 0.014 ± 0.010 | 0.016 ± 0.006 | 0.014 ± 0.006 |
|  | Quinic acid | 31.81 ± 13.59 | 35.50 ± 8.337 | 31.65 ± 11.09 | 26.93 ± 14.77 |
|  | Gluconic acid | 0.076 ± 0.036 | 0.110 ± 0.044 | 0.072 ± 0.066 | 0.175 ± 0.042 |
|  | **Total** | **35.41 ± 12.78** | **38.39 ± 7.924** | **34.17 ± 10.72** | **30.00 ± 14.51** |
|  |  |  |  |  |  |
| Phenolic compounds |  |  |  |  |  |
|  | Chlorogenic acid | 1.124 ± 0.221 | 1.125 ± 1.574 | 1.182 ± 1.093 | 1.413 ± 1.578 |
|  |  |  |  |  |  |
| Others |  |  |  |  |  |
|  | Phosphoric acid | 0.247 ± 0.139 | 0.109 ± 0.042 | 0.154 ± 0.172 | 0.067 ± 0.035 |

Data are presented as mean ± standard deviation. Different letters indicate significant differences among treatments (*P* < 0.05; Kruskal–Wallis test followed by Dunn’s post hoc test with Benjamini–Hochberg correction). Absence of letters denotes non-significant differences (*P* > 0.05). GC–MS, gas chromatography coupled to mass spectrometry. C, control (*n* = 5); D, drought (*n* = 5); O, ozone (*n* = 5); DO, combined drought and ozone (*n* = 5).

**Table S7** Relative abundance (%) of nonpolar phase metabolites detected by GC-MS in leaf extracts of *Eugenia uniflora* seedlings exposed to drought and ozone stresses, alone or in combination

| **Chemical family** | **Putative**  **annotation** | **Treatments** | | | |
| --- | --- | --- | --- | --- | --- |
|  |  | **C** | **D** | **O** | **DO** |
| Sesquiterpenes |  |  |  |  |  |
|  | Elemene | 0.033 ± 0.049 | 0.013 ± 0.009 | 0.031 ± 0.023 | 0.020 ± 0.015 |
|  | β-Elemene | 0.058 ± 0.039 | 0.048 ± 0.043 | 0.086 ± 0.034 | 0.051 ± 0.035 |
|  | β-Caryophyllene | 0.054 ± 0.072 | 0.050 ± 0.041 | 0.049 ± 0.042 | 0.054 ± 0.044 |
|  | γ-Elemene | 0.168 ± 0.271 | 0.125 ± 0.126 | 0.119 ± 0.036 | 0.106 ± 0.095 |
|  | α-Guaiene | 0.060 ± 0.023 | 0.053 ± 0.053 | 0.038 ± 0.033 | 0.049 ± 0.016 |
|  | β-Gurjunene | 0.023 ± 0.016 | 0.016 ± 0.010 | 0.028 ± 0.023 | 0.014 ± 0.005 |
|  | γ-Muurolene | 0.061 ± 0.059 | 0.033 ± 0.019 | 0.083 ± 0.075 | 0.038 ± 0.016 |
|  | δ-Selinene | 0.056 ± 0.019 | 0.046 ± 0.034 | 0.058 ± 0.042 | 0.045 ± 0.018 |
|  | δ-Guaijene | 0.525 ± 0.540 | 0.533 ± 0.490 | 1.516 ± 1.393 | 0.742 ± 0.558 |
|  | γ-Cadinene | 0.008 ± 0.008 | 0.014 ± 0.024 | 0.010 ± 0.014 | 0.002 ± 0.002 |
|  | σ-Cadinene | 0.047 ± 0.035 | 0.050 ± 0.055 | 0.062 ± 0.058 | 0.038 ± 0.019 |
|  | Sesquiterpene 1 | 0.020 ± 0.020 | 0.038 ± 0.067 | 0.024 ± 0.024 | 0.017 ± 0.012 |
|  | Selina-3,7(11)-diene | 0.007 ± 0.006 | 0.018 ± 0.032 | 0.012 ± 0.011 | 0.008 ± 0.006 |
|  | Germacrene B | 0.113 ± 0.196 | 0.082 ± 0.082 | 0.081 ± 0.025 | 0.070 ± 0.064 |
|  | Spathulenol | 0.041 ± 0.043 | 0.027 ± 0.029 | 0.094 ± 0.117 | 0.032 ± 0.030 |
|  | Globulol | 0.032 ± 0.022 | 0.029 ± 0.039 | 0.023 ± 0.027 | 0.024 ± 0.009 |
|  | β-Elemenone | 0.036 ± 0.032 | 0.096 ± 0.101 | 0.108 ± 0.121 | 0.138 ± 0.202 |
|  | β-Atlantol | 0.613 ± 1.074 | 0.657 ± 0.904 | 0.528 ± 0.875 | 0.672 ± 0.602 |
|  | α-Cyperone | 0.053 ± 0.057 | 0.197 ± 0.203 | 0.211 ± 0.272 | 0.264 ± 0.431 |
|  | Sesquiterpene 2 | 0.003 ± 0.006 | 0.052 ± 0.106 | 0.003 ± 0.004 | 0.001 ± 0.000 |
|  | Germazone | 0.065 ± 0.110 | 0.039 ± 0.054 | 0.055 ± 0.078 | 0.064 ± 0.061 |
|  | Sesquiterpene 3 | 0.175 ± 0.106 | 0.190 ± 0.080 | 0.237 ± 0.105 | 0.215 ± 0.046 |
|  | Sesquiterpene 4 | 0.038 ± 0.072 | 0.001 ± 0.001 | 0.001 ± 0.001 | 0.022 ± 0.048 |
|  | Sesquiterpene 5 | 0.150 ± 0.245 | 0.231 ± 0.298 | 0.197 ± 0.279 | 0.190 ± 0.185 |
|  | **Total** | **2.438 ± 2.374** | **2.637 ± 1.787** | **3.653 ± 1.651** | **2.876 ± 1.029** |
|  |  |  |  |  |  |
| Diterpenes |  |  |  |  |  |
|  | Neophytadiene | 0.677 ± 0.067 | 0.743 ± 0.208 | 0.780 ± 0.253 | 0.776 ± 0.265 |
|  | Phytol | 0.020 ± 0.004 | 0.021 ± 0.004 | 0.023 ± 0.006 | 0.018 ± 0.003 |
|  | **Total** | **0.697 ± 0.068** | **0.764 ± 0.211** | **0.804 ± 0.258** | **0.794 ± 0.266** |
|  |  |  |  |  |  |
| Triterpenes |  |  |  |  |  |
|  | Squalene | 0.125 ± 0.036 | 0.177 ± 0.100 | 0.215 ± 0.063 | 0.203 ± 0.108 |
|  | Oleanolic acid | 0.093 ± 0.018 | 0.036 ± 0.038 | 0.457 ± 0.414 | 0.016 ± 0.012 |
|  | Ursolic acid | 0.242 ± 0.134 | 0.297 ± 0.239 | 0.116 ± 0.085 | 0.296 ± 0.163 |
|  | Triterpene 1 | 1.000 ± 0.296 | 0.566 ± 0.105 | 0.792 ± 0.318 | 0.808 ± 0.333 |
|  | Betulin | 0.803 ± 0.212 | 0.323 ± 0.147 | 0.685 ± 0.303 | 0.482 ± 0.356 |
|  | **Total** | **2.264 ± 0.353** | **1.399 ± 0.537** | **2.264 ± 0.818** | **1.805 ± 0.693** |
|  |  |  |  |  |  |
| Phytosterols |  |  |  |  |  |
|  | Phytosterol 1 | 0.074 ± 0.023 | 0.087 ± 0.030 | 0.096 ± 0.030 | 0.076 ± 0.027 |
|  | Phytosterol 2 | 0.102 ± 0.038 | 0.118 ± 0.044 | 0.127 ± 0.038 | 0.108 ± 0.045 |
|  | Phytosterol 3 | 0.397 ± 0.116 | 0.488 ± 0.177 | 0.514 ± 0.158 | 0.423 ± 0.150 |
|  | Phytosterol 4 | 0.092 ± 0.066 | 0.116 ± 0.060 | 0.176 ± 0.136 | 0.113 ± 0.098 |
|  | Stigmasterol | 1.716 ± 0.114 | 1.706 ± 0.258 | 1.896 ± 0.429 | 1.654 ± 0.158 |
|  | β-Sitosterol | 0.122 ± 0.041 | 0.166 ± 0.147 | 0.081 ± 0.024 | 0.261 ± 0.255 |
|  | **Total** | **2.503 ± 0.345** | **2.681 ± 0.497** | **2.891 ± 0.767** | **2.635 ± 0.404** |
|  |  |  |  |  |  |
| Hydrocarbons |  |  |  |  |  |
|  | n-Pentadecane | 0.026 ± 0.004 | 0.022 ± 0.006 | 0.025 ± 0.005 | 0.024 ± 0.003 |
|  | Hydrocarbon 1 | 0.040 ± 0.004 | 0.045 ± 0.007 | 0.035 ± 0.021 | 0.045 ± 0.004 |
|  | Hydrocarbon 2 | 0.108 ± 0.049 | 0.102 ± 0.043 | 0.167 ± 0.102 | 0.135 ± 0.045 |
|  | **Total** | **0.174 ± 0.048** | **0.168 ± 0.049** | **0.228 ± 0.111** | **0.204 ± 0.047** |
|  |  |  |  |  |  |
| Fatty acids |  |  |  |  |  |
|  | Fatty acid 1 | 0.207 ± 0.140 | 0.173 ± 0.114 | 0.167 ± 0.165 | 0.210 ± 0.042 |
|  | Fatty acid 2 | 0.664 ± 0.591 | 0.638 ± 0.423 | 0.607 ± 0.595 | 0.773 ± 0.149 |
|  | Palmitic acid | 6.192 ± 0.562 | 5.512 ± 0.879 | 7.391 ± 1.342 | 5.860 ± 0.709 |
|  | Margaric acid | 0.041 ± 0.017 | 0.031 ± 0.010 | 0.052 ± 0.023 | 0.028 ± 0.007 |
|  | Linoleic acid | 0.777 ± 0.102 | 0.748 ± 0.160 | 0.960 ± 0.209 | 0.691 ± 0.108 |
|  | Linolenic acid | 5.748 ± 1.020 | 6.059 ± 2.768 | 8.370 ± 1.256 | 6.033 ± 2.030 |
|  | Stearic acid | 2.107 ± 0.250 | 1.766 ± 0.156 | 2.150 ± 0.176 | 1.821 ± 0.164 |
|  | Arachidic acid | 0.075 ± 0.006 | 0.089 ± 0.067 | 0.089 ± 0.020 | 0.068 ± 0.007 |
|  | Behenic acid | 0.029 ± 0.011 | 0.042 ± 0.031 | 0.034 ± 0.014 | 0.029 ± 0.012 |
|  | Lignoceric acid | 0.031 ± 0.004 | 0.044 ± 0.037 | 0.041 ± 0.013 | 0.036 ± 0.020 |
|  | **Total** | **17.146 ± 1.376** | **16.376 ± 3.999** | **21.107 ± 1.869** | **16.896 ± 3.040** |
|  |  |  |  |  |  |
| Glycerolipids |  |  |  |  |  |
|  | Glycerol-3-phosphate | 0.160 ± 0.032 | 0.146 ± 0.045 | 0.193 ± 0.030 | 0.145 ± 0.028 |
|  | 1-Monomyristin | 0.408 ± 0.014 | 0.417 ± 0.055 | 0.373 ± 0.082 | 0.417 ± 0.021 |
|  | 2-Monopalmitin | 1.275 ± 0.106 | 1.273 ± 0.235 | 1.245 ± 0.238 | 1.345 ± 0.104 |
|  | 1-Monopalmitin | 28.503 ± 2.431 | 30.547 ± 2.089 | 26.110 ± 5.487 | 29.075 ± 2.010 |
|  | 2-Monostearin | 0.938 ± 0.091 | 0.917 ± 0.139 | 0.909 ± 0.205 | 0.993 ± 0.102 |
|  | 1-Monostearin | 21.548 ± 1.670 | 22.554 ± 1.564 | 19.618 ± 4.314 | 21.700 ± 1.444 |
|  | **Total** | **51.557 ± 4.033** | **54.582 ± 3.749** | **47.203 ± 10.055** | **52.329 ± 3.437** |
|  |  |  |  |  |  |
| Others |  |  |  |  |  |
|  | Phosphoric acid | 0.317 ± 0.024 | 0.286 ± 0.026 | 0.328 ± 0.049 | 0.284 ± 0.035 |
|  | (7Z,11Z)-Hexadecandien-1-ol | 0.203 ± 0.018 | 0.217 ± 0.057 | 0.225 ± 0.048 | 0.238 ± 0.065 |
|  | Quininic acid | 0.078 ± 0.044 | 0.020 ± 0.032 | 0.040 ± 0.032 | 0.012 ± 0.016 |
|  | Mannitol | 0.322 ± 0.209 | 0.039 ± 0.025 | 0.391 ± 0.678 | 0.035 ± 0.010 |
|  | (+-)-α-Tocopherol | 0.464 ± 0.093 | 0.499 ± 0.234 | 0.634 ± 0.259 | 0.595 ± 0.226 |

Data are presented as mean ± standard deviation. Different letters indicate significant differences among treatments (*P* < 0.05; Kruskal–Wallis test followed by Dunn’s post hoc test with Benjamini–Hochberg correction). Absence of letters denotes non-significant differences (*P* > 0.05). GC–MS, gas chromatography coupled to mass spectrometry. C, control (*n* = 5); D, drought (*n* = 5); O, ozone (*n* = 5); DO, combined drought and ozone (*n* = 5).

**Table S8** Summary of statistical analyses for selected polar and nonpolar metabolites with large global and pairwise effect sizes in *Eugenia uniflora* seedlings exposed to drought and ozone stresses, alone or in combination

| Compound | χ^2^ (df) | Kruskal-Wallis  *p*-value | η^2^[H] | Global magnitude | Comparison | VDA | Peer-to-peer magnitude |
| --- | --- | --- | --- | --- | --- | --- | --- |
| **POLAR** |  |  |  |  |  |  |  |
| Proline | 7.18 (3) | 0.325 | 0.261 | Large | C x D | 0.84 | Large |
|  |  |  |  |  | C x O | 0.92 | Large |
|  |  |  |  |  | C x DO | 0.84 | Large |
|  |  |  |  |  | D x O | 0.56 | Small |
|  |  |  |  |  | D x DO | 0.24 | Large |
|  |  |  |  |  | O x DO | 0.28 | Large |
| Sucrose | 7.46 (3) | 0.317 | 0.279 | Large | C x D | 0.28 | Large |
|  |  |  |  |  | C x O | 0.12 | Large |
|  |  |  |  |  | C x DO | 0.04 | Large |
|  |  |  |  |  | D x O | 0.32 | Medium |
|  |  |  |  |  | D x DO | 0.24 | Large |
|  |  |  |  |  | O x DO | 0.36 | Medium |
| Glycerol | 7.83 (3) | 0.298 | 0.302 | Large | C x D | 0.04 | Large |
|  |  |  |  |  | C x O | 0.32 | Medium |
|  |  |  |  |  | C x DO | 0.12 | Large |
|  |  |  |  |  | D x O | 0.76 | Large |
|  |  |  |  |  | D x DO | 0.36 | Medium |
|  |  |  |  |  | O x DO | 0.20 | Large |
| 5-oxoproline | 8.19 (3) | 0.298 | 0.324 | Large | C x D | 0.44 | Small |
|  |  |  |  |  | C x O | 0.08 | Large |
|  |  |  |  |  | C x DO | 0.08 | Large |
|  |  |  |  |  | D x O | 0.20 | Large |
|  |  |  |  |  | D x DO | 0.16 | Large |
|  |  |  |  |  | O x DO | 0.36 | Medium |
| Erythronic acid | 5.38 (3) | 0.510 | 0.149 | Large | C x D | 0.28 | Large |
|  |  |  |  |  | C x O | 0.32 | Medium |
|  |  |  |  |  | C x DO | 0.00 | Large |
|  |  |  |  |  | D x O | 0.56 | Small |
|  |  |  |  |  | D x DO | 0.36 | Medium |
|  |  |  |  |  | O x DO | 0.32 | Medium |
| Tartaric acid | 8.17 (3) | 0.298 | 0.323 | Large | C x D | 0.12 | Large |
|  |  |  |  |  | C x O | 0.08 | Large |
|  |  |  |  |  | C x DO | 0.12 | Large |
|  |  |  |  |  | D x O | 0.16 | Large |
|  |  |  |  |  | D x DO | 0.36 | Medium |
|  |  |  |  |  | O x DO | 0.56 | Small |
| Gluconic acid | 11.16 (3) | 0.298 | 0.510 | Large | C x D | 0.04 | Large |
|  |  |  |  |  | C x O | 0.48 | None |
|  |  |  |  |  | C x DO | 0.00 | Large |
|  |  |  |  |  | D x O | 0.64 | Medium |
|  |  |  |  |  | D x DO | 0.00 | Large |
|  |  |  |  |  | O x DO | 0.12 | Large |
| *Myo*-inositol | 5.31 (3) | 0.510 | 0.144 | Large | C x D | 0.28 | Large |
|  |  |  |  |  | C x O | 0.24 | Large |
|  |  |  |  |  | C x DO | 0.12 | Large |
|  |  |  |  |  | D x O | 0.40 | Small |
|  |  |  |  |  | D x DO | 0.16 | Large |
|  |  |  |  |  | O x DO | 0.44 | Small |
| Lactose | 6.59 (3) | 0.388 | 0.224 | Large | C x D | 0.28 | Large |
|  |  |  |  |  | C x O | 0.12 | Large |
|  |  |  |  |  | C x DO | 0.04 | Large |
|  |  |  |  |  | D x O | 0.44 | Small |
|  |  |  |  |  | D x DO | 0.36 | Medium |
|  |  |  |  |  | O x DO | 0.28 | Large |
| Raffinose | 10.21 (3) | 0.298 | 0.451 | Large | C x D | 0.08 | Large |
|  |  |  |  |  | C x O | 0.04 | Large |
|  |  |  |  |  | C x DO | 0.04 | Large |
|  |  |  |  |  | D x O | 0.32 | Medium |
|  |  |  |  |  | D x DO | 0.20 | Large |
|  |  |  |  |  | O x DO | 0.36 | Medium |
| Polyol 2 | 5.31 (3) | 0.510 | 0.144 | Large | C x D | 0.24 | Large |
|  |  |  |  |  | C x O | 0.08 | Large |
|  |  |  |  |  | C x DO | 0.20 | Large |
|  |  |  |  |  | D x O | 0.48 | None |
|  |  |  |  |  | D x DO | 0.28 | Large |
|  |  |  |  |  | O x DO | 0.36 | Medium |
| Sugar 3 | 7.88 (3) | 0.298 | 0.305 | Large | C x D | 0.16 | Large |
|  |  |  |  |  | C x O | 0.28 | Large |
|  |  |  |  |  | C x DO | 0.08 | Large |
|  |  |  |  |  | D x O | 0.56 | Small |
|  |  |  |  |  | D x DO | 0.16 | Large |
|  |  |  |  |  | O x DO | 0.16 | Large |
| Sugar 6 | 8.39 (3) | 0.298 | 0.337 | Large | C x D | 0.24 | Large |
|  |  |  |  |  | C x O | 0.52 | None |
|  |  |  |  |  | C x DO | 0.08 | Large |
|  |  |  |  |  | D x O | 0.76 | Large |
|  |  |  |  |  | D x DO | 0.16 | Large |
|  |  |  |  |  | O x DO | 0.08 | Large |
| Sugar 10 | 10.27 (3) | 0.298 | 0.454 | Large | C x D | 0.76 | Large |
|  |  |  |  |  | C x O | 1.00 | Large |
|  |  |  |  |  | C x DO | 1.00 | Large |
|  |  |  |  |  | D x O | 0.80 | Large |
|  |  |  |  |  | D x DO | 0.72 | Large |
|  |  |  |  |  | O x DO | 0.36 | Medium |
| Sugar 11 | 5.65 (3) | 0.510 | 0.166 | Large | C x D | 0.44 | Small |
|  |  |  |  |  | C x O | 0.40 | Small |
|  |  |  |  |  | C x DO | 0.12 | Large |
|  |  |  |  |  | D x O | 0.32 | Medium |
|  |  |  |  |  | D x DO | 0.12 | Large |
|  |  |  |  |  | O x DO | 0.24 | Large |
| Sugar 13 | 8.98 (3) | 0.298 | 0.374 | Large | C x D | 0.16 | Large |
|  |  |  |  |  | C x O | 0.20 | Large |
|  |  |  |  |  | C x DO | 0.00 | Large |
|  |  |  |  |  | D x O | 0.52 | None |
|  |  |  |  |  | D x DO | 0.20 | Large |
|  |  |  |  |  | O x DO | 0.20 | Large |
| Total sugars | 8.34 (3) | 0.316 | 0.334 | Large | C x D | 0.28 | Large |
|  |  |  |  |  | C x O | 0.12 | Large |
|  |  |  |  |  | C x DO | 0.00 | Large |
|  |  |  |  |  | D x O | 0.36 | Medium |
|  |  |  |  |  | D x DO | 0.20 | Large |
|  |  |  |  |  | O x DO | 0.32 | Medium |
| **NONPOLAR** |  |  |  |  |  |  |  |
| n-Pentadecane | 5.35 (3) | 0.837 | 0.147 | Large | C x D | 0.88 | Large |
|  |  |  |  |  | C x O | 0.68 | Medium |
|  |  |  |  |  | C x DO | 0.40 | Small |
|  |  |  |  |  | D x O | 0.28 | Large |
|  |  |  |  |  | D x DO | 0.16 | Large |
|  |  |  |  |  | O x DO | 0.32 | Medium |
| Quininic acid | 7.43 (3) | 0.723 | 0.277 | Large | C x D | 0.88 | Large |
|  |  |  |  |  | C x O | 0.72 | Large |
|  |  |  |  |  | C x DO | 0.92 | Large |
|  |  |  |  |  | D x O | 0.24 | Large |
|  |  |  |  |  | D x DO | 0.48 | None |
|  |  |  |  |  | O x DO | 0.80 | Large |
| Mannitol | 6.43 (3) | 0.770 | 0.214 | Large | C x D | 0.88 | Large |
|  |  |  |  |  | C x O | 0.68 | Medium |
|  |  |  |  |  | C x DO | 0.80 | Large |
|  |  |  |  |  | D x O | 0.20 | Large |
|  |  |  |  |  | D x DO | 0.36 | Medium |
|  |  |  |  |  | O x DO | 0.80 | Large |
| Palmitic acid | 8.98 (3) | 0.723 | 0.374 | Large | C x D | 0.92 | Large |
|  |  |  |  |  | C x O | 0.36 | Medium |
|  |  |  |  |  | C x DO | 0.40 | Small |
|  |  |  |  |  | D x O | 0.00 | Large |
|  |  |  |  |  | D x DO | 0.08 | Large |
|  |  |  |  |  | O x DO | 0.52 | None |
| Margaric acid | 7.43 (3) | 0.723 | 0.277 | Large | C x D | 0.92 | Large |
|  |  |  |  |  | C x O | 0.44 | Small |
|  |  |  |  |  | C x DO | 0.68 | Medium |
|  |  |  |  |  | D x O | 0.04 | Large |
|  |  |  |  |  | D x DO | 0.28 | Large |
|  |  |  |  |  | O x DO | 0.72 | Large |
| Stearic acid | 7.71 (3) | 0.723 | 0.294 | Large | C x D | 0.92 | Large |
|  |  |  |  |  | C x O | 0.60 | Small |
|  |  |  |  |  | C x DO | 0.52 | None |
|  |  |  |  |  | D x O | 0.08 | Large |
|  |  |  |  |  | D x DO | 0.08 | Large |
|  |  |  |  |  | O x DO | 0.44 | Small |
| Oleanolic acid | 13.58 (3) | 0.215 | 0.661 | Large | C x D | 0.88 | Large |
|  |  |  |  |  | C x O | 0.12 | Large |
|  |  |  |  |  | C x DO | 1.00 | Large |
|  |  |  |  |  | D x O | 0.04 | Large |
|  |  |  |  |  | D x DO | 0.60 | Small |
|  |  |  |  |  | O x DO | 1.00 | Large |
| Ursolic acid | 5.31 (3) | 0.837 | 0.144 | Large | C x D | 0.48 | None |
|  |  |  |  |  | C x O | 0.80 | Large |
|  |  |  |  |  | C x DO | 0.28 | Large |
|  |  |  |  |  | D x O | 0.72 | Large |
|  |  |  |  |  | D x DO | 0.28 | Large |
|  |  |  |  |  | O x DO | 0.12 | Large |
| Betulin | 6.47 (3) | 0.770 | 0.217 | Large | C x D | 1.00 | Large |
|  |  |  |  |  | C x O | 0.76 | Large |
|  |  |  |  |  | C x DO | 0.64 | Medium |
|  |  |  |  |  | D x O | 0.16 | Large |
|  |  |  |  |  | D x DO | 0.36 | Medium |
|  |  |  |  |  | O x DO | 0.60 | Small |
| Sesquiterpene 4 | 5.48 (3) | 0.837 | 0.155 | Large | C x D | 0.88 | Large |
|  |  |  |  |  | C x O | 0.84 | Large |
|  |  |  |  |  | C x DO | 0.80 | Large |
|  |  |  |  |  | D x O | 0.34 | Medium |
|  |  |  |  |  | D x DO | 0.52 | None |
|  |  |  |  |  | O x DO | 0.64 | Medium |
| Triterpene 1 | 6.22 (3) | 0.770 | 0.201 | Large | C x D | 0.92 | Large |
|  |  |  |  |  | C x O | 0.76 | Large |
|  |  |  |  |  | C x DO | 0.56 | Small |
|  |  |  |  |  | D x O | 0.24 | Large |
|  |  |  |  |  | D x DO | 0.16 | Large |
|  |  |  |  |  | O x DO | 0.40 | Small |

BVOC: biogenic volatile organic compound. C: control. D: drought. O: ozone. DO: drought + ozone. χ² (df): chi-square statistic (degrees of freedom). Differences among groups were evaluated using the Kruskal–Wallis test, followed by Dunn’s post hoc test with Benjamini–Hochberg (BH) correction for multiple comparisons (*P* < 0.05 was considered statistically significant). Global effect sizes were estimated using eta-squared (η²[H]), with thresholds of 0.01, 0.06, and 0.14 indicating small, medium, and large effects, respectively (Cohen, 1988; Lakens, 2013). Pairwise effect sizes were estimated using Vargha and Delaney’s A (VDA), where values range from 0 to 1 and 0.5 indicates no effect. Values > 0.5 indicate higher values in the first group of the comparison, whereas values < 0.5 indicate higher values in the second group. Effect magnitude was interpreted based on the distance from 0.5 (|VDA − 0.5|), with thresholds of 0.56, 0.64, and 0.71 corresponding to small, medium, and large effects, respectively (Vargha and Delaney, 2000).

**Data availability**

GNPS – Polar phase

Deconvolution workflow:

<https://gnps.ucsd.edu/ProteoSAFe/status.jsp?task=d8880f084ee347dc8652134f84326031>

Molecular networking and analysis:

<https://gnps.ucsd.edu/ProteoSAFe/status.jsp?task=673d139d26164604afa1dfab1c230e25>

GNPS – Nonpolar phase

Deconvolution workflow:

<https://gnps.ucsd.edu/ProteoSAFe/status.jsp?task=6c56bfc9121a41729475a9eca3adc930>

Molecular networking and analysis:

<https://gnps.ucsd.edu/ProteoSAFe/status.jsp?task=be643afd7c81463286c74d670f3d2b95>
